# Human iPSC-Derived Neuronal Cells From *CTBP1*-Mutated Patients Reveal Altered Expression of Neurodevelopmental Gene Networks

**DOI:** 10.3389/fnins.2020.562292

**Published:** 2020-10-27

**Authors:** S. Vijayalingam, Uthayashanker R. Ezekiel, Fenglian Xu, T. Subramanian, Elizabeth Geerling, Brittany Hoelscher, KayKay San, Aravinda Ganapathy, Kyle Pemberton, Eric Tycksen, Amelia K. Pinto, James D. Brien, David B. Beck, Wendy K. Chung, Christina A. Gurnett, G. Chinnadurai

**Affiliations:** ^1^Department of Molecular Microbiology and Immunology, Saint Louis University School of Medicine, Edward A. Doisy Research Center, St. Louis, MO, United States; ^2^Department of Clinical Health Sciences, Doisy College of Health Science, Saint Louis University School of Medicine, Saint Louis, MO, United States; ^3^Department of Biology and Henry and Amelia Nasrallah Center for Neuroscience, Saint Louis University, St. Louis, MO, United States; ^4^McDonnell Genome Institute, Washington University School of Medicine, St. Louis, MO, United States; ^5^National Human Genome Research Institute, National Institutes of Health, Bethesda, MD, United States; ^6^Department of Pediatrics and Medicine, Columbia University Medical Center, New York, NY, United States; ^7^Department of Neurology, Washington University School of Medicine, St. Louis, MO, United States

**Keywords:** transcriptional repression, CtBP, *de novo* mutation, interferon response, intellectual and developmental disabilities, transcriptome analysis

## Abstract

A recurrent *de novo* mutation in the transcriptional corepressor *CTBP1* is associated with neurodevelopmental disabilities in children (Beck et al., 2016, 2019; Sommerville et al., 2017). All reported patients harbor a single recurrent *de novo* heterozygous missense mutation (p.R342W) within the cofactor recruitment domain of CtBP1. To investigate the transcriptional activity of the pathogenic *CTBP1* mutant allele in physiologically relevant human cell models, we generated induced pluripotent stem cells (iPSC) from the dermal fibroblasts derived from patients and normal donors. The transcriptional profiles of the iPSC-derived “early” neurons were determined by RNA-sequencing. Comparison of the RNA-seq data of the neurons from patients and normal donors revealed down regulation of gene networks involved in neurodevelopment, synaptic adhesion and anti-viral (interferon) response. Consistent with the altered gene expression patterns, the patient-derived neurons exhibited morphological and electrophysiological abnormalities, and susceptibility to viral infection. Taken together, our studies using iPSC-derived neuron models provide novel insights into the pathological activities of the *CTBP1* p.R342W allele.

## Introduction

The C-terminal Binding Protein (CtBP) family consists of two highly related paralogs, CtBP1 and CtBP2 (and their splice forms) in vertebrates (Chinnadurai, 2007). The nuclear isoforms of CtBP1 (CtBP1-L, NM_001328.2) and CtBP2 (CtBP2-L, NM_022802.2) function as transcriptional corepressors (reviewed in Chinnadurai, 2007). CtBPs mediate transcriptional repression by targeting various chromatin-modifying enzymes to the promoter regions and by interacting with DNA-bound repressors. CtBPs bind with the chromatin modifying factors and various repressors through a high-affinity protein-binding interface known as PXDLS-binding cleft. In addition, an auxiliary protein-binding interface termed RRT-binding groove in CtBPs is involved in interaction with certain Zinc-finger-containing transcription factors. The CtBP1 corepressor complex mediates coordinated histone modifications by deacetylation and methylation of histone H3K9 and demethylation of histone H3K4 (Shi et al., 2003). CtBPs also activate transcription under certain specific contexts (Fang et al., 2006; Paliwal et al., 2012; Bajpe et al., 2013; Itoh et al., 2013; Ray et al., 2014). Since CtBPs are NAD(H)-binding proteins (Kumar et al., 2002; Nardini et al., 2003), the intracellular levels of NAD(H) dinucleotides differentially regulate their transcriptional activity through oligomerization (Zhang et al., 2002).

Studies on mice with disruptions in the *ctbp* genes, showed that *ctbp1* and *ctbp2* play overlapping and unique transcriptional roles during development (Hildebrand and Soriano, 2002). While homozygous deletion of the *ctbp2* gene was embryonic lethal affecting brain and heart development, homozygous deletion of c*tbp1* resulted in viable mice with reduced size and lifespan. In humans, overexpression of *CTBP1* and *CTBP2* was reported in a number of epithelial cancers and was associated with transcriptional activity that leads to epithelial to mesenchymal transformation (reviewed by Chinnadurai, 2002; Byun and Gardner, 2013; Dcona et al., 2017). A role of *CTBP1* in human neurodevelopment was revealed with the discovery of a recurrent *de novo* missense mutation in *CTBP1* (c.991C → T, p.R331W in NM_001012614.1; p.R342W c.1024 C → T in NM_001328.2) in patients with neurodevelopmental features including intellectual disability, ataxia, hypotonia, as well as tooth enamel defects (Beck et al., 2016, 2019; Sommerville et al., 2017). The neurodevelopmental phenotypes conferred by the *CTBP1* mutant allele provide genetic evidence that *CTBP1* is important for normal human brain development. Previous biochemical studies also suggested that CtBPs might be important for certain brain developmental functions (Sahu et al., 2017; Shen et al., 2017). CtBP1 has been reported to mediate transcriptional repression of a number of neuronal genes involved in synaptic activities of the inner ear hair cells, the retina (Ivanova et al., 2015) and the synaptic ribbon complex (Tom Dieck et al., 2005).

The mechanism by which the pathogenic *CTBP1* mutant allele contributes to neurodevelopmental disease is not known. The mutation (referred here as p.R342W) maps within an α-helical region (α-5) of CtBP1 that forms a part of the PXDLS-protein interaction cleft. In a glioblastoma cell line with exogenously expressed *CTBP1* p.R342W, the interactions of various CtBP-cofactors were reduced with the mutant protein (Beck et al., 2019). In order to determine the altered transcriptional profiles in patient-derived cell models, we generated iPSCs from dermal fibroblasts, differentiated them into early stage neurons, and determined their transcriptional profiles by RNA-seq. The morphological and physiological changes inferred from the altered gene expression profiles of patient-derived cells were also determined. Here, we report that genes involved in neurodevelopment, adhesion and antiviral-response pathways are downregulated in *CTBP1* heterozygous p.R342W neurons. Consistent with the transcriptome data, patient-derived heterozygous p.R342W neurons also showed morphological and physiological abnormalities and susceptibility to neurotropic viral pathogenesis.

## Results

### Neuronal Cell Models

**FIGURE 1:**
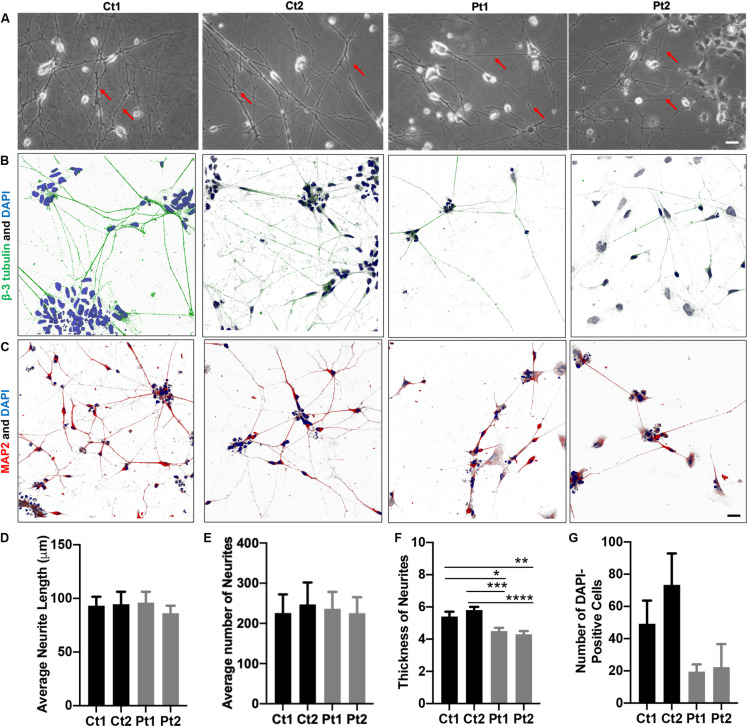
Morphology of iPSC/NSC-derived neurons. Neurite networks of healthy donor control (Ct1 and Ct2) and patient (Pt1 and Pt2) neurons were revealed by phase contrast imaging **(A)** and immunofluorescence imaging of cells stained with antibodies to cytoskeletal proteins beta-3-Tubulin **(B)** and MAP2 **(C)**. The length **(D)**, number **(E)** and thickness **(F)** of neurites were measured from phase contrast images and quantified. There were no significant differences in either the number or the length of neurites between control or patient-cells. Specifically, the average neurite length **(D)** of different cells was: Ct1, 93.2 ± 8.4 μm; Ct2, 94.6 ± 11.6 μm; Pt1, 96.1 ± 10.1 μm; and Pt2, 86.2 ± 7.0 μm (*p* = 0.882; *n* = 5). The average number **(E)** of neurites per image was: Ct1, 225.8 ± 46.4; Ct 2, 247.2 ± 54.6: Pt1, 236.0 ± 42.4; and Pt2, 225.4 ± 39.8 (*p* = 0.985; *n* = 5). The average neurite thickness **(F)** in Ct1 was 5.4 ± 0.3 μm, Ct2 was 5.8 ± 0.2 μm, Pt1 was 4.5 ± 0.2 μm, and Pt2 was 4.3 ± 0.2 μm (*p* = 0.03 for Ct1 vs Pt1; p = 0.00045 for Ct2 vs Pt1; *p* = 0.005 for Ct1 vs Pt2; *p* = 0.00004 for Ct2 vs Pt2; *n* = 10 primary neurites measured in 5 phase contrast images for all conditions, totaling 50 neurites measured per condition). * Indicates a significance level of *p* < 0.05, **p < 0.01, ***p < 0.001, and ****p < 0.0001. **(G)** Number of DAPI-Positive Cells Bar Graph. DAPI staining of nuclei revealed fewer patient neurons adhered to the culture dish. Number of DAPI-positive cells in Ct1 was 49.2 ± 14.4, in Ct2 was 73.4 ± 19.5, in Pt1 was 19.5 ± 4.5, and in Pt2 was 22.3 ± 14.3 (*p* = 0.0507; *n* = 6, 6, 5, and 3 fluorescent images analyzed for Ct1, Pt1, Ct2, and Pt2, respectively). Scale bar is 25 μm.

Since the *CTBP1* p.R342W mutation is associated with neurodevelopmental disabilities, we designed experiments to compare the transcriptional profiles of patient and healthy control derived neuronal cell models. We generated iPSCs from the dermal fibroblasts of two patients with the *CTBP1* p.R342W heterozygous mutation and two age-matched normal donors using Sendai virus delivery of the Yamanaka factors (Klf-4, Sox-2, Oct3/4, and c-Myc) (Ban et al., 2011; Nayler et al., 2017). We further differentiated the iPSC (Supplementary Figure 1A) into neural stem cells (NSC) (Supplementary Figure 1B) and early (14-days of differentiation) neurons (Figure 1) using neural differentiation media. These neurons were used for transcriptional profiling and morphological and electrophysiological comparisons.

The phase contrast images of iPSC/NSC-derived neurons of normal donors and patients revealed typical neuronal morphology, exhibiting ability to grow and extend robust neurites and form inter- connections (Figure 1). To quantify the differences in neurite length and number, phase contrast images were measured using the ImageJ software, NeuronJ (Meijering et al., 2004; Pemberton et al., 2018). While there were no significant differences between patient and control cells in these outgrowth parameters (Figures 1D,E), analysis of neurite thickness demonstrated a significantly decreased primary neurite thickness in patient cells compared to control cells (Figure 1A, arrows; Figure 1F). The neurons were also analyzed by immunocytochemistry labeling of the cytoskeletal markers beta-3 Tubulin (green) (Figure 1B) and Microtubule-Associated Protein 2 (MAP2, red) (Figure 1C). This analysis confirmed robust and extensive internetworks formed by both control- and patient-derived neurons. We also quantified the staining of nuclei with DAPI (blue). Compared to controls, patient cultures had, in general, fewer DAPI-positive cells (Figure 1G). Together, these results indicated that neurons derived from *CTBP1*-mutated stem cells survived less in culture. Although they were able to form and extend neurites, like control neurons, the neurites of patient neurons were significantly thinner.

### Transcriptomic Profiling of iPSC/NSC-Derived Neurons

**FIGURE 2:**
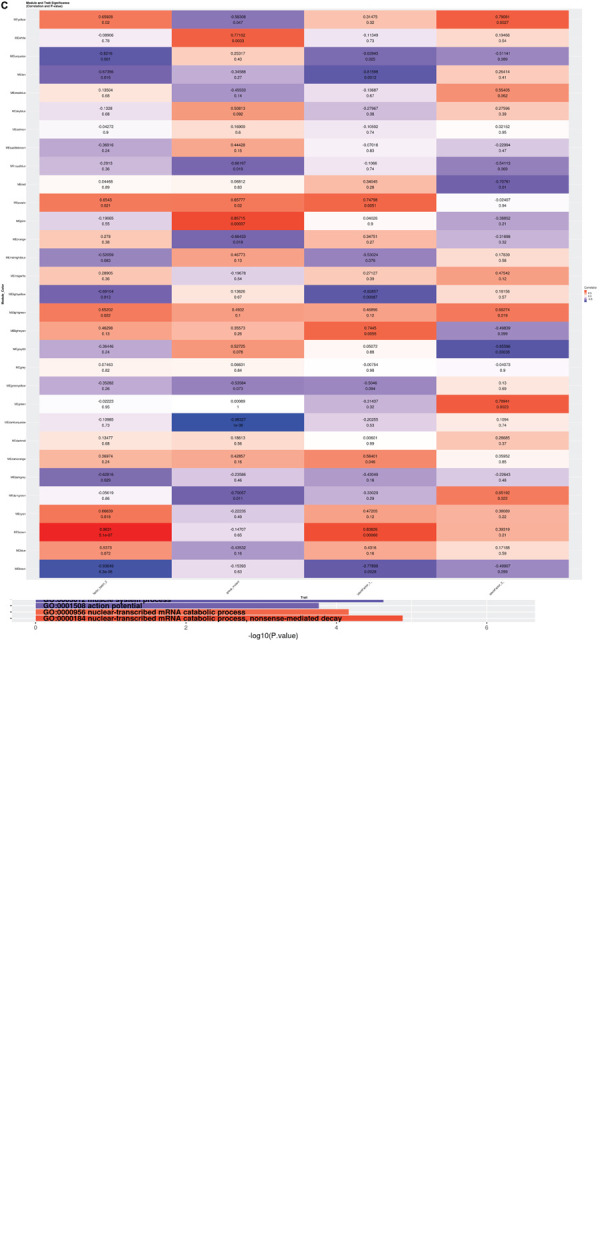
**(A)** Volcano Plot: Volcano plot of 15,942 gene transcripts with log 2 fold changes observed by Limma. 7,141 were differentially expressed between patient and control neurons (FDR ≤ 0.05). Among these transcripts, 6,500 genes were protein-coding. Out of these, 161 were down-regulated (Blue) and 36 were up-regulated (Red) by 3-fold or more. **(B)** Global GO Biological Process Perturbation Bar Plot: All 15,942 transcripts were interrogated by Gage for level perturbations across all known GO biological process gene sets. The significance and mean log 2 fold change of each term was evaluated by *t*-tests. **(C)** Module and Trait Eigengene Correlation and Significance Matrix: Matrix of *de novo* color coded modules found by WGCNA and their respective eigengene Pearson correlation (top value in each cell) and p-values (bottom value in each cell) for the mutation and statistical covariates. Bright red modules are high positively correlated and bright deep blue are high negatively correlated transcripts. Modules with absolute value Pearson correlations greater than 75% to the mutant samples (pink, white, and darkturquoise) were considered the best candidates for further investigation.

To determine the transcriptional profiles altered by the *CTBP1* mutant allele (p.R342W) in patient-derived cell models, we prepared RNA from the neurons (14-days after differentiation) generated from two different patient-derived NSC and two healthy donor NSC lines. The cDNA generated from RNA derived from the early neurons were sequenced on an Illumina HiSeq 3000 with single-end 50 base pair reads. The sequence reads were aligned with STAR (Dobin et al., 2013), and were quantitated with Subread (Liao et al., 2014). The gene counts were analyzed using established methods for quantifying gene expression: the R/Bioconductor package Limma (Ritchie et al., 2015) and SVA (Leek et al., 2012). Our analysis revealed that out of 15,942 gene transcripts robustly expressed at greater than 1 count-per-million in at least 5 samples, 7,141 were differentially expressed between patient and control neurons (FDR ≤ 0.05). Among these transcripts, 6,500 genes were protein-coding. As depicted in the volcano plot, among the differentially expressed protein-coding transcripts, 161 were down-regulated and 36 were up-regulated by 3-fold or more in patient-derived neurons (Figure 2A). All genes were then tested for perturbations in gene ontology (GO) biological processes (Figure 2B).

**FIGURE 3:**
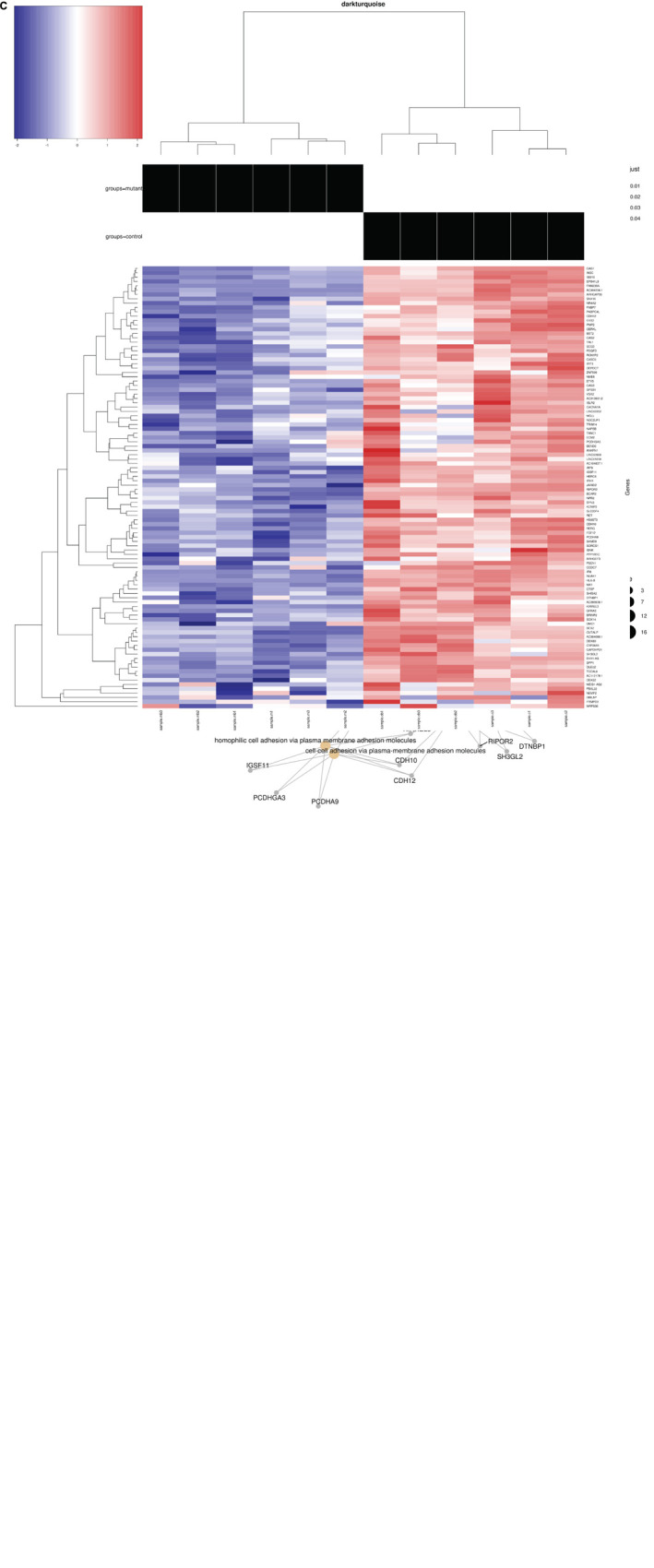
GO network plot for down-regulated genes and heat maps: **(A)** Darkturquoise GO Biological Process Enrichment Bar Plot. All 102 negatively correlated WGCNA *de novo* clustered darkturquoise genes were tested for enrichment across known GO biological processes with the R/Bioconductor package clusterProfiler. The color illustrates the significance of the enrichment and the size of each bar characterizes the number of genes for each enriched term. The down regulation and negative correlation of darkturquoise module genes alludes to the down regulation represented by each significant term. **(B)** GO Category Network Plot: The negatively correlated genes clustered into a WGCNA *de novo* network labeled as darkturquoise were tested for enrichment across known GO biological processes with the R/Bioconductor package clusterProfiler. Significant terms with overlapping genes are clustered together while the overlapping genes represented in the darkturquoise network module illustrate the connections between those terms. **(C)** Heat map: Heat map representing 102 genes in the darkturquoise WGCNA module whose z-scores illustrate their down regulation and high negative correlation across all mutant samples.

The differentially expressed genes were then subjected to Weighted Gene Correlation Network Analysis (WGCNA) (Langfelder and Horvath, 2008). A matrix of *de novo* color-coded modules found by WGCNA and correlated with the mutation are shown in Figure 2C. The modules with high correlations (Pearson correlations >75%) to *CTBP1* p.R342W mutant cells were selected for further analysis; darkturquoise (Figure 3), pink (Supplementary Figure 2) and white (Supplementary Figure 3). The correlation of the eigengenes for every cluster revealed a highly negative correlated module labeled in darkturquoise containing 102 transcripts and highly positively correlated modules labeled in pink (451 transcripts) and white (78 transcripts). GO enrichment analysis of the darkturquoise module revealed highly significant down regulation of genes involved in neuronal development/functions, synaptic cell adhesion, and type 1 interferon signaling and response (Figures 3A,B). A heat-map of these highly correlated genes confirmed that the genes associated with these biological processes were down-regulated across all *CTBP1* mutant samples (Figure 3C). The genes that were significantly up-regulated in patient cells in the pink cluster (Supplementary Figures 2A–C) appear to be involved in diverse biological processes such as protein synthesis and protein targeting/localization, RNA catabolic process and apoptosis signaling. The white module of up-regulated genes in *CTBP1* mutant cells were significantly enriched for metabolic processes, transcriptional initiation and translation as shown in Supplementary Figures 3A–C.

**FIGURE 4:**
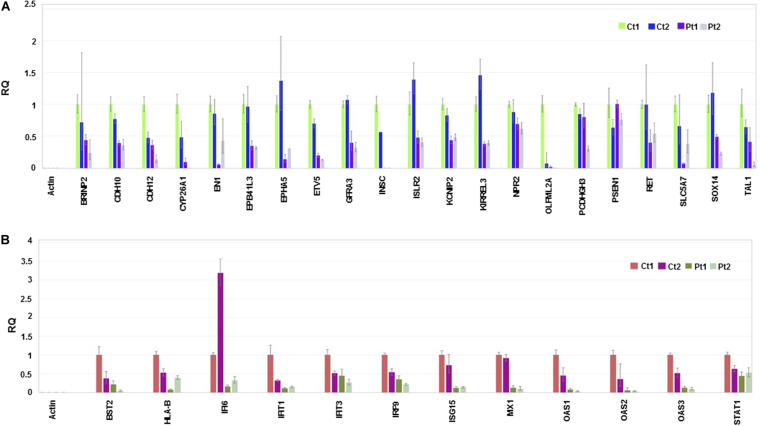
RT-qPCR validation. The experiment was carried out in triplicate and the relative quantification (RQ) values are plotted and the data analysis done in Thermo Fisher Connect^TM^ (Cloud) https://www.thermofisher.com/us/en/home/digital-science.html. Endogenous and reference control used in the experiment is Actin and control (Ct1), respectively. **(A)** Relative quantification of genes involved in neuronal development and cell adhesion. **(B)** Relative quantification of genes involved in Interferon responses.

Considering the neuronal developmental phenotypes, including intellectual disability exhibited by patients with the *CTBP1* p.R342W mutation and adherence phenotypes of the mutant cells observed while differentiating NSC to neurons (not shown), we pursued RNA sequencing and observed the suppression of genes involved in neuronal development and cell adhesion (Figure 4A and Supplementary Table 1). We then focused on the genes of down-regulated biological processes for validation by RT-qPCR analysis (Figure 4). The down-regulated transcriptional pattern was prominent in the transcriptome data analysis and the expression patterns of a number of transcripts were readily validated by RT-qPCR analysis. Further, the known functions of several down-regulated genes appeared to be relevant to the patient phenotypes (see Supplementary Table 2). In contrast to the down-regulated transcripts, up-regulated transcripts were diverse (see below). The down-regulated genes included those involved in neuronal development and cell adhesion (Figure 4A and Supplementary Table 1). In agreement with the RNA-seq data sets, RT-qPCR analysis revealed that several genes involved in type I interferon-response were also repressed in patient neurons (Figure 4B and Supplementary Table 1). In contrast to the negatively correlated and down-regulated genes in darkturquoise, the more variable heatmaps and lower statistical significance of genes in the positively correlated up-regulated pink and white clusters were diverse. Query with RT-qPCR analysis of several up-regulated genes suggested that these clusters did not merit further pursuit (data not shown). However, the possibility of up-regulation of isolated genes (none identified here) cannot be ruled out.

### Physiological and Biological Activities of *CTBP1* p.R342W-Mutated Neurons

**FIGURE 5:**
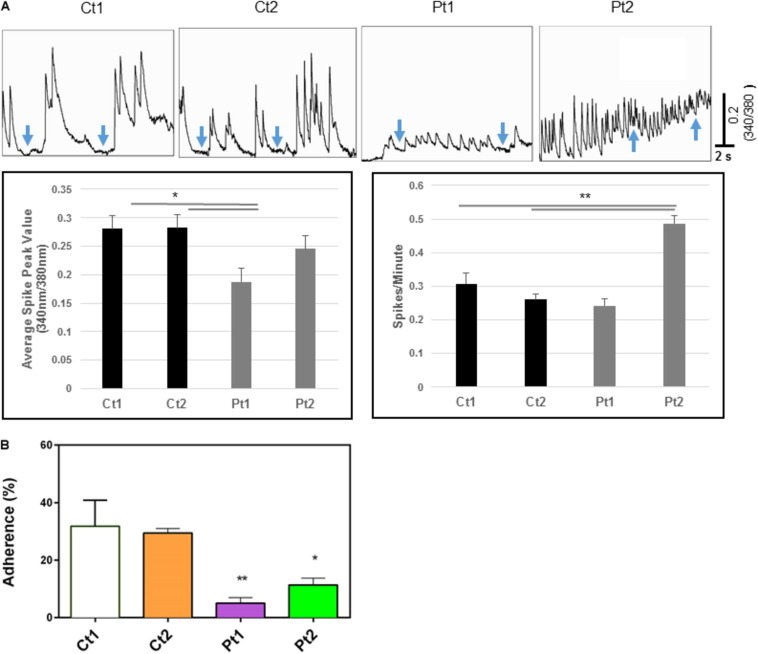
**(A)** Calcium imaging data. Top panels show raw traces of spontaneous calcium transients recorded from neurons of control donors and patients. Bottom bar graphs are quantitative data of the mean peak amplitudes and spiking frequency of calcium transients. Our data show that neurons from patients (Pt1 and Pt2) exhibited significant changes in either the amplitude or the frequency of calcium transients with the inability to return to basal levels compared to their matched donor controls (Ct1 and Ct2). Arrows indicate basal levels between calcium transcients. **(B)** Adhesion activity of neurons. Control (Ct1 and Ct2) and patient-derived (Pt1 and Pt2) neurons (6-days after differentiation from NSC) were plated onto Geltrex coated 8-well glass slide Chamber and incubated at 37°C for 30 min, the dishes were flipped to remove non-adherent cells. The adherent neurons were stained with an antibody specific to MAP2 and DAPI and MAP2-expressing cells were counted. Percentage of adherent (flipped) cells was calculated based on un-flipped cells. Patient-derived cells exhibited statistically less adhesion than control cells Ct1 and Ct2: Pt1 (*p* < 0.05) and Pt2 (<0.01). *n* = 3.

**Calcium transients:** The above transcriptome analysis revealed down-regulation of neurodevelopmental and interferon response genes, we carried out additional assays to examine whether *CTBP1* p.R342W mutation affects factors such as cytosolic calcium (Ca^2+^) levels and plasma membrane ion currents that are involved in normal neuronal functions. It is well known that brief and repetitive elevations of intracellular calcium levels (spontaneous calcium transients) are important in regulating various neural developmental processes, including neural survival, differentiation, neurite outgrowth, synaptic transmission and plasticity (Spitzer et al., 1994; Spitzer, 2006; Rosenberg and Spitzer, 2011). To assess the effect of *CTBP1* p.R342W on calcium transients, we performed fluorescent calcium imaging experiments on control donor and patient neurons. Our results revealed that neurons derived from patients exhibited differences in the frequency and/or amplitude of Ca^2+^ transients as compared to control neurons (Figure 5). Specifically, both control neurons (Ct1 and Ct2) exhibited regular compound patterns of calcium oscillations, and showed regular spiking activity with the ability to return to the baseline between spikes (indicated by blue arrows in Figure 5, left two panels). Interestingly, the patient neurons (Pt1 and Pt2) showed either more sustained elevation of intracellular Ca^2+^ with significantly reduced amplitude (*p* < 0.05, Figure 5) or more frequent, irregular patterns of Ca^2+^ transients with the calcium levels rarely returning to baseline (indicated by blue arrows in Figure 5, right two panels). These results indicate that *CTBP1* p.R342W impacts internal Ca^2+^ oscillations, either affecting their amplitudes or spiking patterns leading to dysregulation of Ca^2+^ homeostasis in patient neurons. We note that in spite of the inter-patient variations between the two patient-derived cell lines (Pt1 and Pt2), they both exhibited consistent irregular Ca^2+^ transients. It is possible that the effect of *CTBP1*-mutation might be additionally influenced by other stochastic intra-patient environments.

Because spontaneous Ca^2+^ transients are normally driven by active firing of neuronal action potentials mediated by the functional expression of inward sodium (Na^+^) and outward potassium (K^+^) currents, we next examined whether the *CTBP1* p.R342W mutation alters these currents in neurons. We specifically focused on the impact of *CTBP1* p.R342W on the voltage-gated Na^+^ currents, which are essential for the ability of neurons to generate action potentials. Our whole cell patch clamping data (Supplementary Figure 4) showed that neurons from control donors exhibited normal expression of Na^+^ currents. However, the neurons derived from one patient (Pt1) expressed little or no Na^+^ currents in all neurons examined and the neurons derived from the second patient (Pt2) exhibited either smaller or much larger Na^+^ currents (Supplementary Figure 4). As in the case Ca^2+^ transient measurements, both Pt1 and Pt2 cells showed inter-patient variations in Na^+^ current measurements. Taken together, our data suggest that the *CTBP1* p.R342W mutation may affect the normal neuronal functions such as intracellular Ca^2+^ homeostasis and membrane excitability, two fundamental factors that mediate neuronal communications in the nervous system.

**Adhesion activities:** While performing routine cell culture procedures, we observed that *CTBP1*-mutated cells exhibited increased ability to become detached from the culture surface as individual cells when treated with cell detachment agents. Our transcriptome analysis revealed down-regulation of several adhesion molecules involved in cell-cell and synaptic adhesion (Figure 3B and Supplementary Table 2). To experimentally determine whether the *CTBP1* p.R342W neurons exhibit less adhesion activities, we carried out a “flipping” assay (Langhe et al., 2016). The control and patient cells were plated onto Geltrex coated chamber slides and incubated. After short culture time, the adherent and non-adherent cells were determined by flipping one set of plates. The cells in both flipped and un-flipped plates were fixed and stained with DAPI and MAP2-antibody. The percentage of adhered (flipped) cells was calculated based on un-flipped cells. We found that the percent of adherent cells were statistically lower in patient-derived neurons. As shown in Figure 5B, the patient-derived neurons exhibited significantly less adhesion than controls (Pt1-p < 0.05; Pt2-p < 0.01). As cadherins function in cell-cell contacts, reduced adherence of patient neurons suggest that the expression of these molecules may be affected. This *in vitro* result correlates with our transcriptome analysis where several cadherins (CDH10, CDH12 and KRREL3) are down-regulated (Supplementary Table 2).

**FIGURE 6:**
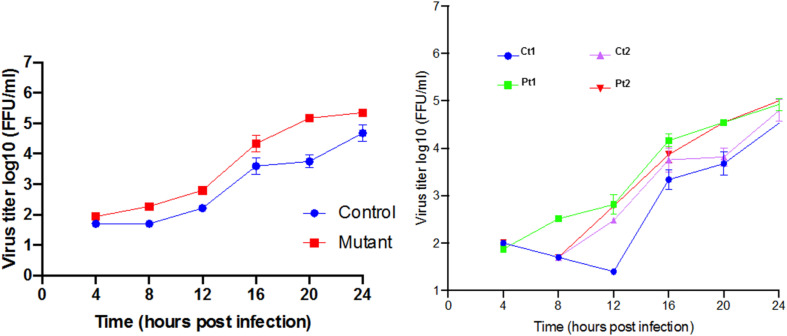
Single step growth curves for WNV replication in neurons: The single step growth curves were generated by determining the viral titers at different times after infection in triplicates. The media supernatants of infected cells were collected at indicated times during 24 h of infection and stored at –80°C prior to viral titer analysis by focus forming assay. The average mean (±SD) of controls (Ct1 and Ct2) and patient cells (Pt1 and Pt2) are on the left. The values for the individual control and patient cells are on the right.

**Response to neurotropic virus infection:** Our transcriptome analysis revealed prominent down regulation of homeostatic levels of interferon-stimulated genes. West Nile Virus infection is known to directly infect neurons both in mice (Shrestha et al., 2003) and in humans (Diamond et al., 2009) and can cause neuronal injury by direct cytopathic effect. Multiple studies have demonstrated that WNV is highly susceptible to antiviral interferon stimulated genes (ISG), which can act to reduce viral titer and alter cell susceptibility (Jiang et al., 2010). Therefore, we hypothesized that *CTBP1* p.R342W neuronal cells would be more susceptible to WNV replication as compared to healthy control neurons. To test this, we performed a single step growth curve using WNV on both patient and healthy control-derived neurons (Figure 6). We noted that there was a higher level of WNV replication in the patient-derived *CTBP1* p.R342W neuronal cells as compared to healthy control neurons from 4 h post infection until the end of the assay. These results were statistically significant at 20 h post infection (*p* ≤ 0.01). We interpret these results to mean that the *CTBP1* p.R342W neurons might be more susceptible to WNV infection as the virus replicated to higher titers in mutant neurons compared to control neurons for most of the time points tested (about 100-fold at 20 h after infection). These results are consistent with the levels of expression of various anti-viral response genes in the patient neurons.

## Discussion

A specific *W342* mutation in the transcriptional corepressor *CTBP1* leads to prominent neurodevelopmental manifestations including intellectual disability, ataxia, and hypotonia in affected patients (Beck et al., 2016, 2019; Sommerville et al., 2017). The neurodevelopmental phenotypes conferred by the *CTBP1* p.R342W allele provide genetic evidence that *CTBP1* is important for normal human neurodevelopment. We employed patient iPSC-derived neuronal cell models to determine the transcriptional activities of the pathogenic *CTBP1* p.R342W allele and the potential link to functional abnormalities in neurons. By using iPSC-derived “early” neurons of two different patients with *CTBP1* p.R342W mutations (out of 12 known patients) and two healthy age-matched donors, we determined the transcriptional profiles through RNA-seq analysis. These results revealed down-regulation of genes involved in three major neuronal functions: including cell adhesion, neurodevelopment, and antiviral (type I interferon) response (Figure 3). The altered gene expression profiles appear to be consistent with intellectual and movement disorder phenotypes seen in patients with *CTBP1* mutations (Beck et al., 2016, 2019; Sommerville et al., 2017).

Our results revealed down-regulation of several neuronal adhesion genes such as *KIRREL3*, *CDH10* and *CDH12* (Figure 4A and Supplementary Table 1). Reduced expressions of these genes or specific mutations in these genes have previously been implicated in intellectual disabilities (see citations in Supplementary Table 2). For example, the intellectual disability gene *KIRREL3* was shown to regulate target-specific mossy fiber synapse development in the hippocampus, and cadherin 12 (CDH12) was shown to mediate calcium dependent cell-cell adhesion. The neuronal adhesion genes appear to exert their effects through alterations of synaptic adhesion of neurons. Similarly, the expression of several genes involved in neurodevelopment (e.g., *SOX14*), ion channel activities (e.g., *BRINP2* and *KCNIP2*) and neuronal receptors (*GFRA3*, *NR4A2*, *RET* and *ISLR2*) is also reduced in our patient-derived neurons. Mice lacking *Brinp2* were reported to exhibit neurodevelopmental phenotypes while *Kcnip2* and associated subunits were shown to regulate homeostatic neuronal excitability (ref. in Supplementary Table 2). Additionally, the expressions of certain neuronal transcription factors that are involved in neuronal development and survival (*ETV5*, *TAL1* and *EN1*) were also reduced in patient neurons. These results suggest that the neurodevelopmental phenotypes caused by the *CTBP1* p.R342W allele may be related to global down regulation of a number of genes that are involved in neuron survival, growth, membrane excitability, synaptic transmission and plasticity.

Here, we have used physiologically relevant patient-derived and age-matched control neuronal cell models to determine the transcriptional profiles of primary iPSC-derived neurons. Many human neuronal diseases (including *CTBP1*p.R342W-mutated developmental defects; our unpublished data) do not appear to manifest phenotypes in the mouse models. In the lack of suitable animal models, human neuronal cell models are most relevant. However, we acknowledge the limitations of our study. It involved cells derived from a limited number of patients (2 out of 12 known patients that were available) and these cell models exhibited inherent inter-patient differences. However, application of suitable data analysis approaches revealed a prominent patient-centric transcriptional pattern that was validated by RT-qPCR analysis. We note that the transcriptional patterns of iPSC-derived patient cell models is somewhat different from that of a reconstituted exogenously introduced *CTBP1*p.R342W mutant allele (Beck et al., 2019), suggesting that the cell type (glioblastoma vs primary neuron) and/or the endogenous chromatin context influence the gene expression of the *CTBP1* mutant allele.

Our phase contrast images and immunostaining of DAPI and cytoskeletal β-tubulin and MAP2 markers revealed that patient neurons exhibited thinner neuritic processes and fewer cell bodies as compared to those in control donor neurons. This decrease in DAPI-positive cells in patient cultures could be the result of a decrease in cell survival, proliferation or a change in cell fate. This could be due to the aforementioned compromised cell adhesion genes/proteins in CtBP1 patients’ neurons because adhesion molecules have been reported to play important roles in various neuronal developmental processes including neural precursor cell proliferation, differentiation, growth cone pathfinding, neural excitability, and cell-cell communications (Dihne et al., 2003; Valente et al., 2016). The significant thinner neuritic processes in patients’ neurons may indicate smaller neuritic surface areas for housing ionic channels and transmitter receptors, resulting in the decreased efficiency of neuronal conductivity and synaptic communication. These data suggest that *CTBP1* p.R342W mutation may negatively impact the production of key molecular components of neural cytoskeletal structures. Although no studies have demonstrated a direct link between CtBP1 and the cytoskeleton in mammalian cells, it has been reported that a “CtBP/BARS-like” protein in plants has a direct activity on the microtubule cytoskeleton (Folkers et al., 2002). Specifically, the plant CtBP homolog, Angustifolia (AN) was reported to control polar elongation of leaf cells *via* regulation of microtubule cytoskeleton proteins and mutations in AN caused aberrant development and distribution of the microtubules. Thus, our results along with previously published data suggest that CtBP1 may be important for normal development of neural cytoskeletal structures which in turn contribute to neural morphogenesis and synaptic function. The pathological phenotypes in patients with *CTBP1* p.R342W mutations may be caused by impairments in microtubule development and neuronal connections.

In addition to its roles in neuron morphological development, CtBPs also regulate genes involved in neuronal excitability. For example, CtBPs affect gene expression in epileptogenesis (Hubler et al., 2012; Goldberg and Coulter, 2013; Liu et al., 2017), and are highly expressed in many brain regions where they may play a role in synaptic transmission and plasticity (Tom Dieck et al., 2005; Jose et al., 2008). We showed here that neurons derived from patients with *CTBP1* p.R342W mutations had altered spontaneous Ca^2+^ waves and whole-cell Na^+^ currents, further implying its involvement in neural excitability and synaptic function. Intracellular Ca^2+^ is essential to many developmental events including neural survival, differentiation, proliferation, and neurite outgrowth as well as synapse formation, synaptic transmission, and plasticity (Rosenberg and Spitzer, 2011; Grienberger and Konnerth, 2012). It is becoming increasingly clear that the common pattern of Ca^2+^ signaling in neurons is a pattern of spiking activities (Ca^2+^ transients), and the amplitude and frequency of Ca^2+^ transients are key determinants for normal neuronal development and function (O’Donovan, 1999; Dupont et al., 2011; Gasperini et al., 2017). For example, Ca^2+^ transients but not sustained Ca^2+^ elevations play important roles in axon growth and branching, growth cone turning, and cytoskeletal stabilization in developing mammalian neurons (Tang et al., 2003). Interestingly, our study revealed that Ca^2+^ in neurons derived from *CTBP1*- mutated patients exhibited more sustained patterns, and the amplitude and frequency of Ca^2+^ were significantly altered compared to those in control donor neurons (Figure 5A). Because of the central role of Ca^2+^ in neuronal physiology, even moderate alterations of Ca^2+^ homeostasis may lead to profound functional impairments as shown in several neuronal disorders (Wojda et al., 2008; Kawamoto et al., 2012; Oliveira et al., 2014). Therefore, the *CTBP1* mutation-mediated alteration in Ca^2+^ transients may in turn contribute to the morphological abnormalities in the neuritic cytoskeleton observed in our study. In line with this postulation is a study showing that CtBP1 was a molecular constituent of the subfamily 2 of voltage-gated Ca^2+^ channel (Ca_V_2) proteome in the rat brain, which co-purified with cytoskeletal proteins. These results raised the possibility that CtBP1 may regulate Ca^2+^ signaling *via* Ca_V_ and could play a role in regulating cytoskeletal function. However, future studies are warranted to investigate the exact mechanistic action of CtBP1 on the functions of Ca_V_ and cytoskeletal proteins.

Here, we also provide evidence that *CTBP1* mutation alters whole cell ionic currents including voltage-gated Na^+^ (Na_V_) currents. Na_V_ channels are essential for neuronal electrical activity generation and propagation. The abnormal Na_V_ currents detected in *CTBP1*-mutated neurons in our study suggest that CtBP1 may interact with Na_V_ channels and regulate its expression and/or function in neurons. Furthermore, our RNA-seq data showed that the *CTBP1* p.R342W mutation down regulated KCNIP, a gene encoding a Ca^2+^-binding protein that is an integral subunit component of K_V_4 (Burgoyne, 2007). Activity of K_V_4 currents contributed to neuronal excitability in response to changes in intracellular Ca^2+^ (Burgoyne, 2007). Together these studies provide insights into the involvement of specific ions such as Ca^2+^, Na^+^, or K^+^. Altering the homeostasis of these ions is indicative of changes in action potentials in neurons. However, future studies on direct measurement and comparison of action potentials using isogenic cell models would be of interest to elucidate misregulation of neuronal activities by altered ionic homeostasis.

Brain imaging results have been reported for a subset of patients with *CTBP1* p.R342W mutations and those studies revealed cerebellar volume reduction in consecutive scans (Beck et al., 2016; Sommerville et al., 2017). Among the various human tissues, *CTBP1* is highly expressed in the cerebellum^1^. It was reported that CtBP(1/2) proteins play an anti-apoptotic role in primary cerebellar granule cells as well as in dopaminergic neuron-like cells (Stankiewicz et al., 2013). Our results showed that the patient neurons expressed reduced levels of the homeodomain transcription factor, Engrailed 1 (EN1). The activity of EN1 is required for normal cerebellar differentiation (Wurst et al., 1994; Joyner, 1996) and survival of dopaminergic neurons (Chi et al., 2003; Alvarez-Fischer et al., 2011), suggesting the possibility that reduced expression of EN1 in patients might contribute to the cerebellar pathology of *CTBP1*-mutated patients, hence future investigation of *CTBP1* mutation in cerebellar function is also much wanted.

The effect of *CTBP1* p.R342W on interferon-response genes was unexpected since patients have not demonstrated any increased susceptibility toward infections although this has not been characterized in detail. Our results suggest that the iPSC-derived “early” neurons express constitutive basal levels of type I interferon-response genes and that the expression is diminished in patient-derived “early” neurons. Although the interferon signaling pathway in neurons is not well-studied, homeostatic expression of type I interferon response genes in neurons have been reported (Cavanaugh et al., 2015; Drokhlyansky et al., 2017). Developing neurons respond to pathogenesis by neurotropic viruses *via* production of type I interferon (reviewed by Chakraborty et al., 2010; Nallar and Kalvakolanu, 2014). Since the expression of interferon response genes is lower in *CTBP1*-mutated early neurons compared to the constitutive levels in control cells, our results suggest a role for *CTBP1* in the regulation of interferon response in early neurons. Thus far, a direct role for CtBP1 in regulating the expression of interferon-response genes has not been identified. However, the histone methyltransferase PRDM16 which interacts with CtBPs was reported to repress type I interferon response genes in adipocytes (Kissig et al., 2017) in intestinal epithelium (Stine et al., 2019). PRDM16 also plays critical roles in neuronal development and was previously shown to control embryonic and post-natal neural stem cell maintenance and differentiation in the brain (Inoue et al., 2017; Shimada et al., 2017). It is possible that the *CTBP1* p.R342W allele may augment the activities of repressors such as PRDM16 to reduce the level of constitutive interferon signaling in early neurons. Additionally, it remains to be seen whether peripheral blood from patients demonstrates a similar decrease in interferon-response to neurons. Our results suggest that patients with *CTBP1* mutations may have an additional risk factor of increased susceptibility to neuronal viral pathogens.

**FIGURE 7:**
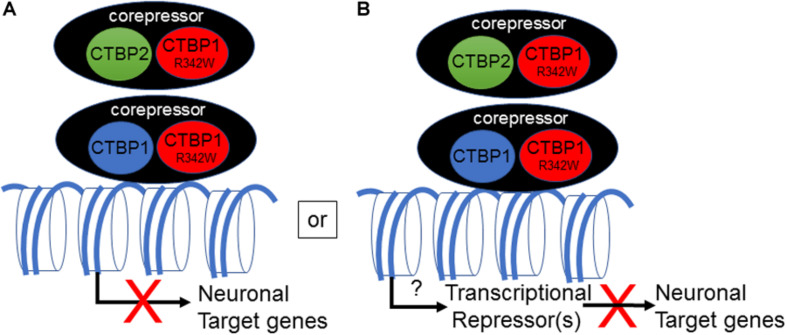
Model for transcriptional down-regulation of neuronal target genes by *CTBP1* mutant allele. CTBP1 mutant protein may homo/heterodimerize with CTBP1 wt or CTBP2 wt. The homo/heterodimeric/oligomeric CTBP-complex may lead to enhanced repression of neuronal target genes as a result of defective dissociation from the target gene promoters directly or indirectly through reduced expression of transcriptional activators **(A)**. Alternatively, the heterodimeric complex may result in up-regulation of a certain transcriptional repressors that may repress a battery of neuronal target genes **(B)**. The down-regulated neurodevelopmental genes function in cell-cell/synaptic adhesion, neuronal transcription, axon growth, ion channel and anti-viral response genes. The transcriptional model does not exclude any potential cytosolic effects of CTBP1 mutant protein indirectly influencing the transcriptional outcome.

The mechanism by which the *CTBP1* p.R342W allele regulates transcription in neuronal cells remains to be determined. All known *CTBP1*-mutated patients contained the same c.C991→T (*CTBP1*-S) transition within the *CTBP1* gene. Since the mutation is heterozygous, it appears that the mutation may either act as a dominant negative or gain of function. An *in silico* prediction suggests potential dominant negative phenotype for the *CTBP1* p.R342W allele (Beck et al., 2016). There have been multiple reports of frame-shift mutations in individuals who are not affected (ExAC database: PMID:27535533), implying that one allele of *CTBP1* is enough to avoid neurologic phenotype. Potential dominant negative activity of the mutant allele may affect functions of both CtBP1 and CtBP2 (Figure 7). It should be noted that Arg at residue 342 is conserved in most vertebrate (including CtBP2) and invertebrate CtBPs, suggesting a pivotal role for it in CtBP functions. The mutation *CTBP1*p.R342W is located within the major protein-interaction cleft (known as PXDLS-binding cleft) that is involved in binding with different transcriptional repression molecules of the CTBP1/2-repression complex (Chinnadurai, 2007), and the mutation was shown to impair such interactions (Beck et al., 2019). Recently, two different *CTBP1*-mutated patients with two different mutations within CTBP1 protein sequences that involved interaction with the components of the CTBP-repression complex have also been identified (David Beck; Nijala Al-Sweel; personal communications). It is possible that the heterozygous *CTBP1* mutant alleles may function as dominant repressors by not dissociating from the target gene promoters (Figure 7). Alternative models may include compromised transcriptional repression activity of the heterozygous *CTBP1* alleles, resulting in relief of repression of a master transactional repressor. Such a repressor may directly repress different neuronal target genes.

Phenotypes caused by the *CTBP1* p.R342W allele partially overlap with some core phenotypes of Wolf-Hirschhorn syndrome (WHS). WHS patients exhibit deletions in the chromosomal region (chromosome 4p16.3) that spans chromosome *CTBP1* locus (Battaglia et al., 2008; Zollino et al., 2008). In certain patients with the smallest micro-deletions in 4p16.3, a cluster of four genes including *NSD2* and *CTBP1* is deleted, implicating *CTBP1* in neurodevelopmental disorders including epilepsy in some WHS patients (Misceo et al., 2012). Thus, it appears that deleting *CTBP1* may contribute to some WHS phenotypes, in addition to mutations in WHS genes. Disrupting *C. elegans* CtBP1 also results in exploration and movement phenotypes (Reid et al., 2015; Yeon et al., 2018). Thus, multiple investigations, including our current transcriptional profiling studies, indicate an emerging importance of CtBP1 in normal neuronal development and activities.

## Materials and Methods

**Fibroblasts:** The human dermal fibroblasts from patients (CSC43 and CSC44, designated here as Pt1 and Pt2, respectively) and healthy donors (LE028 and NT011, designated Ct1 and Ct2, respectively) were received from Columbia University, Department of Pediatrics and Medicine and were grown in DMEM supplemented with 10% fetal bovine serum. Informed consent was obtained from all individual participants included in the study.

**Stem cells:** Patient and donor iPSCs were generated by reprogramming dermal fibroblasts by transduction of Sendai virus (SeV) vectors expressing the Yamanaka factors. Fibroblasts were transduced with CytoTune SeV reprogramming vectors. Eight days after transduction with SeV, colonies were harvested and re-plated on Mouse Embryonic Fibroblast (MEF) culture dishes. The transduced cells were expanded on MEF culture dishes and were then shifted to iPSC medium (ThermoFisher Scientific). The medium was composed of Dulbecco’s Modified Eagle Medium F-12 Mixture (DMEM/F-12), KnockOut Serum Replacement (KnockOut SR), Non-Essential Amino Acids, Basic Fibroblast Growth Factor (FGF-Basic), and 2-mercaptoethanol. Colonies were stained with live cell imaging agent TRA-1-60 Alexa flour 594 conjugate antibody (ThermoFisher Scientific) and picked for further propagation and characterized by immunocytochemistry using iPSC marker, SOX2. In order to convert iPSCs to Neural Stem Cells (NSCs), the cells were plated on Geltrex coated plates and grown with PSC Neural induction media as per the protocol (ThermoFisher Scientific). The differentiated NSC cells were characterized by immunocytochemistry using NSC makers, Nestin.

**Early neurons:** Early neurons were generated by differentiation of NSC. For this, NSCs were first plated on poly-ornithine and laminin coated plates and grown using NSC Serum Free Media (SFM) (ThermoFisher Scientific) for the first 2 days. The NSC SFM was composed of Knockout DMEM/F-12, Stempro Neural Supplement, Recombinant FGF-Basic (Human), GlutaMAX^TM^-I Supplement, and recombinant EGF (Human). After 2 days, they were grown in differentiation medium, which is composed of 1X neurobasal medium, serum free B-27 supplement, and GlutaMAX^TM^-I supplement.

**RNA sequencing and data analysis:** Cells were grown in a 6-well plate. Cell culture media was aspirated and cells were lysed with Trizol (Zymo Research, Irvine, CA, United States). Total RNA was purified using the Direct- Zol RNA kit (Zymo Research, Irvine, CA, United States) following the manufacturer’s protocol. Library preparation was performed with 1 μg of total RNA, concentration was determined by Qubit and integrity was determined using an Agilent tapestation or bioanalyzer. Ribosomal RNA was removed by a hybridization method using Ribo-ZERO kits (Illumina). Depletion and mRNA yield was confirmed by bioanalyzer. mRNA was then fragmented in buffer containing 40 mM Tris acetate pH 8.2, 100 mM potassium acetate and 30 mM magnesium acetate and heating to 94 degrees for 150 s. mRNA was reverse transcribed to yield cDNA using SuperScript III RT enzyme (Life Technologies, per manufacturer’s instructions) and random hexamers. A second strand reaction was performed to yield ds-cDNA. cDNA was blunt ended, had an A base added to the 3’ends, and then had Illumina sequencing adapters ligated to the ends. Ligated fragments were then amplified for 12–15 cycles using primers incorporating unique index tags. Library molarity was determined by Qubit assay for concentration and tapestation for size. An equimolar pool was made of all libraries with unique indices. Fragments were sequenced on an Illumina HiSeq-3000 using single reads extending 50 bases.

Basecalls and demultiplexing were performed with Illumina’s bcl2fastq software and a custom python demultiplexing program with a maximum of one mismatch in the indexing read. RNA-seq reads were then aligned to the Ensembl release 76 top-level assembly with STAR version 2.0.4b. Gene counts were derived from the number of uniquely aligned unambiguous reads by Subread:featureCount version 1.4.5. All gene counts were then imported into the R/Bioconductor package EdgeR and TMM normalization size factors were calculated to adjust for samples for differences in library size. Ribosomal genes and genes not expressed in at least five samples greater than one count-per-million were excluded from further analysis. The TMM size factors and the matrix of counts were then imported into the R/Bioconductor package Limma. Weighted likelihoods based on the observed mean-variance relationship of every gene and samples were then calculated for all samples with the voomWithQualityWeights. Unknown latent effects were estimated with surrogate variable analysis and differential expression analysis was then performed to analyze for differences between conditions and the results were filtered for only those genes with Benjamini-Hochberg false-discovery rate adjusted p-values less than or equal to 0.05. Global log 2 fold-change perturbations in known Gene Ontology (GO) terms and KEGG pathways were detected using the R/Bioconductor package GAGE and deemed significant with Benjamini-Hochberg false-discovery rate adjusted p-value less than or equal to 0.05.

To find the most critical genes, the raw counts were variance stabilized with the R/Bioconductor package DESeq2 and then interrogated via weighted gene correlation network analysis with the R/Bioconductor package WGCNA. Briefly, all genes were correlated across each other by Pearson correlations and clustered by expression similarity into unsigned modules using a power threshold empirically determined from the data. An eigengene was then created for each *de novo* cluster and its expression profile was then correlated across all coefficients of the model matrix. Because these clusters of genes were created by expression profile rather than known functional similarity, the clustered modules were given the names of random colors where gray is the only module that has any pre-existing definition of containing genes that do not cluster well with others. For modules where the eigengene correlation exceeded 75%, the modules of genes were tested for functional enrichment of known GO terms with hypergeometric tests available in the R/Bioconductor package clusterProfiler. Significant terms with Benjamini-Hochberg adjusted *p*-values less than 0.05 were then collapsed by similarity into clusterProfiler category network plots to display the most significant terms for each module of hub genes in order to interpolate the function of each significant module. The hub genes for each significant module were then assessed for whether or not those features were also found to be significantly differentially expressed using Limma.

**Confocal imaging and immunocytochemistry:** Phase contrast images of iPSC/NSC-derived neurons were taken on an inverted microscope (Olympus CKX53). Images were taken under a 20× objective lens and image acquisition parameters were kept consistent between control and patient neurons. After imaging, cells were fixed for 30 min with 4% paraformaldehyde and subsequently washed three times with 1× PBS, permeabilized for 5 min with 0.3% Triton in 1× PBS, and blocked with 5% goat serum diluted in 1× PBS for 1 h. Preparations were then incubated overnight with monoclonal anti-beta-3 Tubulin or anti-MAP2 antibodies produced in mouse (1:500) (Sigma, T0198 for anti-beta-3 Tubulin and Invitrogen, 13-1500 for MAP2). Cells were rinsed three times with 1× PBS the next day. Cells were then incubated with either Alexa Fluor 488 goat anti-mouse IgG secondary antibody (1:500) (ThermoFisher Scientific, A11029) for labeling beta-3 Tubulin or Alexa Fluor 546 goat anti-mouse IgG secondary antibody (1:500) (ThermoFisher Scientific, A11030) for labeling MAP2 for 1 h at room temperature (21–22°C) under dark conditions. Cells were rinsed three times with 1× PBS, and mounted using MOWIOL mounting media with 4′6-diamidino-2-phenylindole dihydrochloride (Sigma, F6057). Samples were acquired and viewed using laser scanning confocal microscopy (Leica TCS SP8 STED 3X super-resolution system) under a 40× oil objective at 488 nm excitation (green, beta-3 Tubulin) with a 515/30 emission filter and 543 nm excitation (red, MAP2) with a 590/50 emission filter. Stack images of 0.7 μm were first collected and compressed into single 3D images. Image acquisition parameters for control and patients’ neurons were kept the same.

To quantify morphological differences between patient and control cells, ImageJ and its plugin NeuronJ were utilized as previously described (Meijering et al., 2004; Pemberton et al., 2018). For neuronal growth parameters (neurite length, number and thickness), phase contrast images were taken of 14-day differentiated neurons as described above. The NeuronJ software was programmed to output length of each neurite (μm) and number of neurites measured in each phase contrast image. For neurite thickness analyses, primary neurites extending directly from a cell body were identified. In each phase contrast image, 10 primary neurites were randomly identified, and the thickest part of the neurite was measured and averaged. To determine differences in the number of DAPI-positive cells between patients and controls, confocal images were analyzed in ImageJ, and the number of DAPI-stained cells was counted. A one-way analysis of variance (ANOVA) was completed to statistically analyze all data followed by Tukey’s HSD *Post hoc* test as appropriate. Data are presented as mean ± SEM.

**Ca^2+^ Imaging:** Ca^2+^ imaging experiments were performed using Fura-2 acetoxymethyl ester (AM) ratiometric Ca^2+^ indicator for monitoring basal intracellular Ca^2+^ levels in iPSC/NSC cells derived from control and patients. Cells differentiated into neurons for about 14 days were loaded with 5 μM Fura-2 AM (ThermoFisher Scientific Cat #: F1201) in HBSS for 30 min at 37°C followed by four 10 min washes in HBSS. Cells sat for 15 min to ensure full conversion of the dye before imaging. Images of each wavelength were taken once every second on an inverted microscope (Olympus IX73) installed with a Retiga R1 camera (Qimage). Excitation wavelengths of 340 and 380 nm were delivered using a LAMBDA XL equipped with high-speed wavelength switcher (Sutter Instrument, Novato, CA, United States) through a 40× objective for 10–20 min. The emitted fluorescence signal was collected at 510 nm by the Retiga R1 camera. Images were acquired with the MetaFluor Imaging software (Olympus) and processed and analyzed using ImageJ. Comparisons were made between individual control and mutant samples using student’s *t*-Test with Benjamini-Hochberg procedure to control for multiple comparisons.

**Whole-Cell Patch-Clamp Recordings:** Whole-cell patch-clamp recordings of voltage-dependent ionic currents were performed on neurons after 14 days of differentiation in culture using a Multiclamp 700B amplifier (Axon Instruments; Sunnyvale, CA, United States) connected to an analog-to-digital interface Digidata 1500A (Axon Instruments). Signals were acquired and stored through pClamp 10.6 software (Axon Instruments). Whole-cell currents were recorded under voltage-clamp mode with the holding potential of −70 mV. Currents were evoked by voltage steps ranging from −90 mV to +60 mV in 10 mV increments. The external solution contained (in mM) NaCl, 135; CaCl_2_, 3; KCl, 5; MgCl_2_, 2; HEPES, 10; D-Glucose, 10; pH adjusted to 7.3 with NaOH. The internal pipette solution was composed of (in mM) CsCl, 130; MgCl_2_, 0.3; HEPES, 10; EGTA, 0.1, ATP-Mg, 3; GTP-Na, 0.6; pH adjusted to 7.3 with CsOH. The osmolarity for internal solution was approximately 300 mOsm (295–305) and for external solution approximately 330 mOsm (320–340). Borosilicate glass pipettes were pulled using a horizontal micropipette puller (Model P-1000, Sutter instrument Co., United States) and had a tip resistance ranging from 3 to 6 MΩ after filling with internal solutions. Only cells with series resistances less than ∼20 MΩ and leaks less than ∼80 pA were selected for the analysis. Traces were processed using Clampfit 10.6 software (Axon Instruments).

**Cell adhesion assay:** The adherence activity of control and *CTBP1-*mutated neurons was determined by a “flipping” assay (Langhe et al., 2016). NSCs were differentiated to neurons (6 days) and dissociated using StemPro Accutase. Cells were counted and plated (1 × 10^4^ cells) onto Geltrex coated Lab-Tek II Chamber 8-well glass slides and incubated for 30 min at 37°C. One set of dishes was flipped over and shaken to remove non-adherent cells. The adherent cells were fixed and stained with DAPI and immunostained with MAP2 antibody. The numbers of adherent cells were determined by counting 3 independent areas for each experiment. The cells were counted using Cytation 3 Cell Imaging Multi-Mode Reader (BioTek). The percentage of adhered (flipped) cells was calculated based on un-flipped cells. Comparisons between control and mutant adherence were performed using one-way analysis of variance (ANOVA) with Bonferroni Multiple Comparisons *post hoc* test. All experimental data were reported as mean ± SEM and three independent experiments were performed. *P* < 0.05 was considered statistically significant.

**West Niles Virus (WNV) replication assay:** WNV-NY (strain 3000.0259) passaged once in Vero cells (African green monkey kidney epithelial cells) was purchased from American Type Culture Collection (ATCC CCL-81). The virus was titered using a standard focus forming assay (FFA) on Vero cells as previously described (Pinto et al., 2014). WNV replication was determined by single step growth in human *CTBP1*-mutated or healthy control neurons. The 14-days differentiated neurons were infected with WNV at MOI = 1 and allowed to incubate for 1 h before the virus was removed. The progeny viral titers were determined by focus forming assay (FFA) as described (Pinto et al., 2014).

## Data Availability Statement

The original contributions presented in the study are publicly available. This data can be found here: https://www.ncbi.nlm.nih.gov/geo/query/acc.cgi?acc=GSE158754.

## Author Contributions

GC designed and directed the project, and wrote the manuscript. SV performed the transcriptome analysis, validation, imaging of neurons and cell adhesion assay. TS participated in the characterization of patient-derived cells. UE generated the stem cells from patient fibroblasts and participated in the transcriptome analysis. KS and AG participated in the generation of stem cells from patient fibroblasts along with UE and FX performed electrophysiological experiments and wrote sections of the manuscript. BH and KP participated in cell and calcium imaging studies. EG, AP, and JB designed the studies on response of neurons to WNV infection. ET performed RNA-seq data analysis. DB, WC, and CG along with GC designed, supervised the project and edited the manuscript. All authors contributed to the article and approved the submitted version.

## Conflict of Interest

The authors declare that the research was conducted in the absence of any commercial or financial relationships that could be construed as a potential conflict of interest.

## Footnotes

1 www.proteinatlas.org/ENSG00000159692-CTBP1/tissue

## Supplementary Material

Supplementary Figure 1 iPSC and NSC immunostaining.

Supplementary Figure 2 GO network plot for positively regulated genes and heat maps. **(A)** GO Biological Process Enrichment Bar Plot (pink): All 454 positively correlated WGCNA *de novo* clustered pink genes were tested for enrichment across known GO biological processes with the R/Bioconductor package clusterProfiler. The color illustrates the significance of the enrichment and the size of each bar characterizes the number of genes for each enriched term. The up-regulation and positive correlation of pink module genes alludes to the up regulation represented by each significant term. **(B)** Module GO Category Network Plot: Positively correlated genes clustered and labeled as pink and tested for enrichment across known GO biological processes with the R/Bioconductor package clusterProfiler. Significant terms with overlapping genes are clustered together while the overlapping genes represented in the pink network module illustrate the connections between those terms. **(C)** Heat map: Heat map representing positively correlated genes in the pink module whose z-scores illustrate their up regulation and high positive correlation across all mutant samples.

Supplementary Figure 3 **(A)** GO Biological Process Enrichment Bar Plot (white): All 81 negatively correlated WGCNA *de novo* clustered white genes were tested for enrichment across known GO biological processes with the R/Bioconductor package clusterProfiler. The color illustrates the significance of the enrichment and the size of each bar characterizes the number of genes for each enriched term. The up-regulation and positive correlation of white module genes alludes to the up-regulation represented by each significant term. **(B)** White Module GO Category Network Plot: All 81 correlated genes clustered into a WGCNA *de novo* network labeled as white were tested for enrichment across known GO biological processes with the R/Bioconductor package clusterProfiler. Significant terms with overlapping genes are clustered together while the overlapping genes represented in the white network module illustrate the connections between those terms. **(C)** White Heat map: Heat map representing 81 genes in the white WGCNA module whose z-scores illustrate their up-regulation and high positive correlation across all mutant samples.

Supplementary Figure 4 Whole-cell patch-clamping. Whole-cell patch-clamp currents were elicited by step depolarizations, and the peak inward currents at −10 mV were analyzed. Left panels show representative raw traces recorded and right panels show the statistical data. Our results, comparing to the matched control donors (Ct1 and Ct2), showed that *CTBP1* mutation either significantly reduced the inward sodium currents in neurons from one patient (Pt1), or induced much larger sodium currents in the other patient (Pt2). Note that Pt2 cells expressed excessively robust inward current which reached to ≥4 nA.

Supplementary Table 1 List of highly down-regulated genes in patient neurons.

Supplementary Table 2 Neurodevelopmental genes down-regulated in *CTBP1*-mutated patient neurons.
