# Supplementary material for: Human iPSC-Derived Neuronal Cells From CTBP1-Mutated Patients Reveal Altered Expression of Neurodevelopmental Gene Networks
Source: Front Neurosci. 2020 Oct 27;14:562292. doi: 10.3389/fnins.2020.562292 (PMC7653094; doi:10.3389/fnins.2020.562292)
Supplement: Supplementary Figure 1 — iPSC and NSC immunostaining. [file Data_Sheet_1.pdf]

ensembl_ID	Gene	Description	logFC	P.Value	adj.P.Valu	transcript_count	module Color
ENSG00000234745	HLA-B	major histocompatibility complex, class I, B	-6.105925	1.50E-13	7.99E-10	10	darkturquoise
ENSG00000188487	INSC	INSC, spindle orientation adaptor protein	-4.250442	8.18E-10	2.81E-07	8	darkturquoise
ENSG00000157601	MX1	MX dynamin like GTPase 1	-3.274864	1.73E-11	2.51E-08	18	darkturquoise
ENSG00000115665	SLC5A7	solute carrier family 5 member 7	-3.151444	5.46E-10	2.48E-07	2	royalblue
ENSG00000111335	OAS2	2'-5'-oligoadenylate synthetase 2	-3.063191	2.19E-07	1.06E-05	7	darkturquoise
ENSG00000089127	OAS1	2'-5'-oligoadenylate synthetase 1	-3.011778	1.70E-09	4.62E-07	11	darkturquoise
ENSG00000187608	ISG15	ISG15 ubiquitin-like modifier	-2.856704	6.49E-14	5.18E-10	3	darkturquoise
ENSG00000163064	EN1	engrailed homeobox 1	-2.769127	2.19E-10	1.51E-07	2	royalblue
ENSG00000111331	OAS3	2'-5'-oligoadenylate synthetase 3	-2.750136	6.17E-10	2.59E-07	7	darkturquoise
ENSG00000185585	OLFML2A	olfactomedin like 2A	-2.686831	2.42E-09	5.68E-07	3	royalblue
ENSG00000130303	BST2	bone marrow stromal cell antigen 2	-2.508783	6.82E-09	1.11E-06	3	darkturquoise
ENSG00000095596	CYP26A1	cytochrome P450 family 26 subfamily A member 1	-2.485809	7.20E-10	2.73E-07	6	darkturquoise
ENSG00000126709	IFI6	interferon alpha inducible protein 6	-2.445744	3.61E-15	5.76E-11	3	darkturquoise
ENSG00000185745	IFIT1	interferon induced protein with tetratricopeptide repeats 1	-2.286596	8.43E-12	1.35E-08	2	royalblue
ENSG00000168875	SOX14	SRY-box 14	-2.276696	1.81E-07	9.32E-06	1	darkturquoise
ENSG00000162367	TAL1	TAL bHLH transcription factor 1, erythroid differentiation factor	-2.204478	1.83E-08	2.09E-06	6	darkturquoise
ENSG00000244405	ETV5	ETS variant 5	-2.196398	4.38E-09	8.12E-07	14	darkturquoise
ENSG00000145242	EPHA5	EPH receptor A5	-1.950151	4.77E-12	1.09E-08	6	blue
ENSG00000119917	IFIT3	interferon induced protein with tetratricopeptide repeats 3	-1.830613	3.20E-09	6.54E-07	2	darkturquoise
ENSG00000154162	CDH12	cadherin 12	-1.792625	1.66E-07	8.74E-06	8	darkturquoise
ENSG00000119922	IFIT2	interferon induced protein with tetratricopeptide repeats 2	-1.602502	6.42E-06	0.00012146	3	darkgreen
ENSG00000146013	GFRA3	GDNF family receptor alpha 3	-1.505017	1.75E-06	4.69E-05	2	darkturquoise
ENSG00000040731	CDH10	cadherin 10	-1.503027	7.77E-10	2.81E-07	4	darkturquoise
ENSG00000082397	EPB41L3	erythrocyte membrane protein band 4.1 like 3	-1.450606	3.65E-11	4.16E-08	36	darkturquoise
ENSG00000167178	ISLR2	immunoglobulin superfamily containing leucine rich repeat 2	-1.288987	2.00E-07	1.01E-05	12	darkturquoise
ENSG00000198797	BRINP2	BMP/retinoic acid inducible neural specific 2	-1.231636	0.0008689	0.00386602	3	darkturquoise
ENSG00000149571	KIRREL3	kirre like nephrin family adhesion molecule 3	-1.220109	2.16E-08	2.34E-06	9	darkturquoise
ENSG00000213928	IRF9	interferon regulatory factor 9	-1.212617	2.35E-06	5.90E-05	15	darkturquoise
ENSG00000120049	KCNIP2	potassium voltage-gated channel interacting protein 2	-1.172459	3.39E-06	7.64E-05	14	darkturquoise
ENSG00000254245	PCDHGA3	protocadherin gamma subfamily A, 3	-1.148602	0.0003724	0.00205432	3	darkturquoise
ENSG00000115415	STAT1	signal transducer and activator of transcription 1	-1.087111	4.88E-10	2.36E-07	12	royalblue
ENSG00000159899	NPR2	natriuretic peptide receptor 2	-0.659034	9.13E-07	2.92E-05	6	darkturquoise
ENSG00000080815	PSEN1	presenilin 1	-0.109012	0.1075402	0.1762206	25	darkturquoise

Supplementary Table 1.

**Transcripts down regulated in patient cells**	**Function**	**Phenotypes in human/animal models**	**Reference**
BRINP2	Neurodevelopment; neural cell proliferation; bone morphogenesis	Behavioral defects in mice	(Berkowicz et al., 2016)
CDH10	Autism-spectrum Disorder-like	Cell Adhesion	(Wang et al., 2009)
CDH12	Schizophrenia	Cell Adhesion	(Redies et al., 2012)
ETV5	Neuronal transcription factor;	Development of hippocampal neurons	(Fontanet et al., 2018) (Williams et al., 2016) (Liu et al., 2016)
GFRA3	Receptor for GFRα3/GDNF	Development of axonal projections	PNAS (Souza et al., 2010) (Schaller et al., 2017)
ISLR2	Receptor for LRR	Forebrain development; axon routing	(Abudureyimu et al., 2018) (Panza et al., 2015)
KCNIP2	Ion channel subunit;	Homeostasis in hippocampal neurons; susceptibility to epilepsy; Ca^{2+} transient	(Wang et al., 2013) (Nassal et al., 2017)
KIRREL3	Cell adhesion; synaptic adhesion	Intellectual disability gene; autism, Jacobsen's syndrome	(Martin et al., 2015) (Bhalla et al., 2008) (Guerin et al., 2012)
NR4A2	Orphan receptor	Parkinson's, Alzheimer's	(Moon et al., 2019)
RET	RET Tyr kinase signaling	Hirschsprung's disease	(Tomuschat et al., 2015)
SOX14	Cerebello-olivary projections	Cerebellar functions	(Prekop et al., 2018)
TAL1	bHLH transcription factor	GABAergic neurogenesis; Post mitotic neuronal features	(Achim et al., 2013; Lee et al., 2017)
NPR2	natriuretic peptide receptor 2	synaptic connections between axons; axon bifurcation	(Lu et al., 2014; Ter-Avetisyan et al., 2018)
DMC1	Rec A homolog	*neuronal* survival and differentiation	(Rivera et al., 2015)
EN1	Homeobox transcription factor	Cerebellar development	(Martinez et al., 2013; Orvis et al., 2012)

Supplementary Table 2

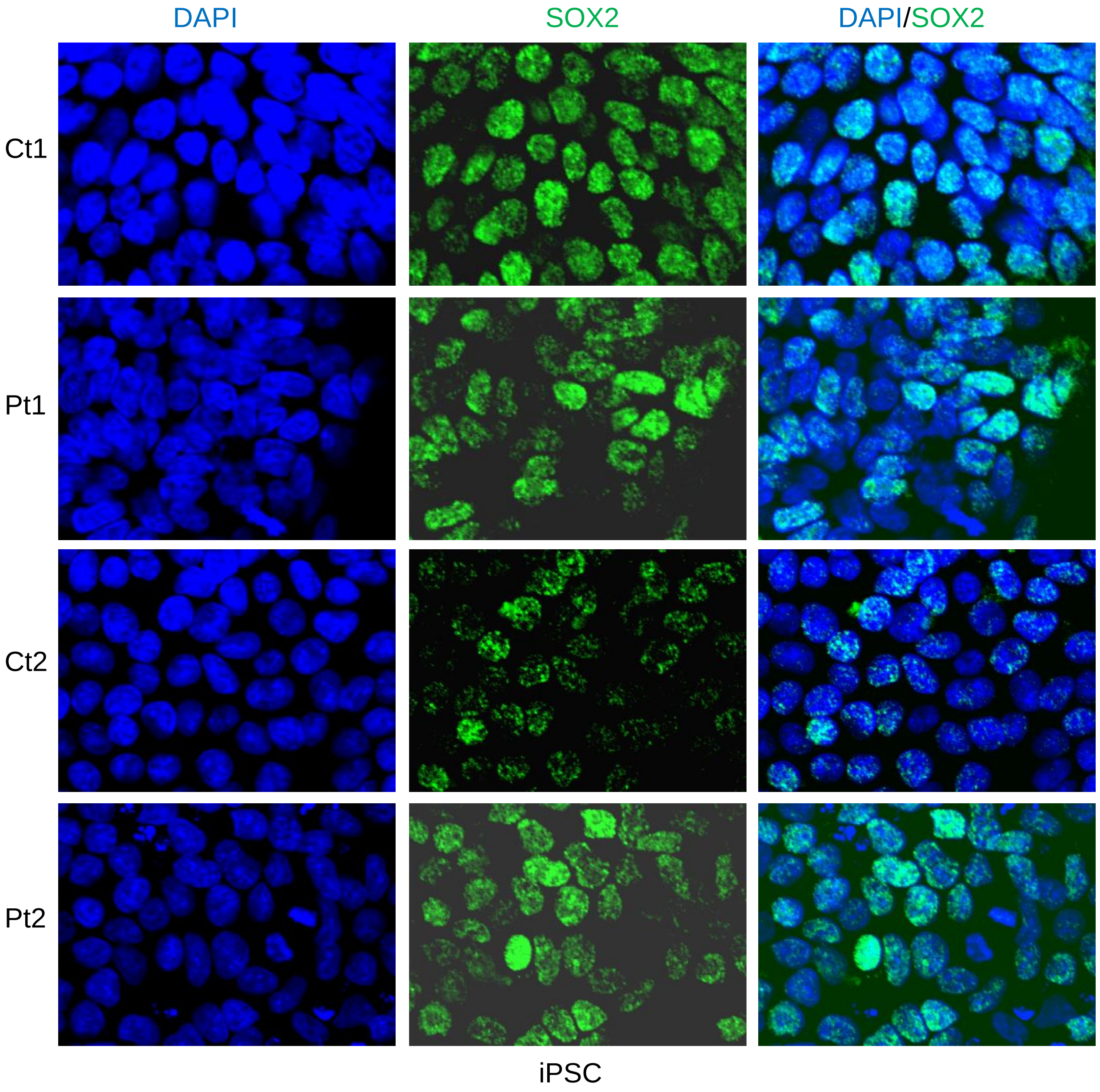

Fig. S1A

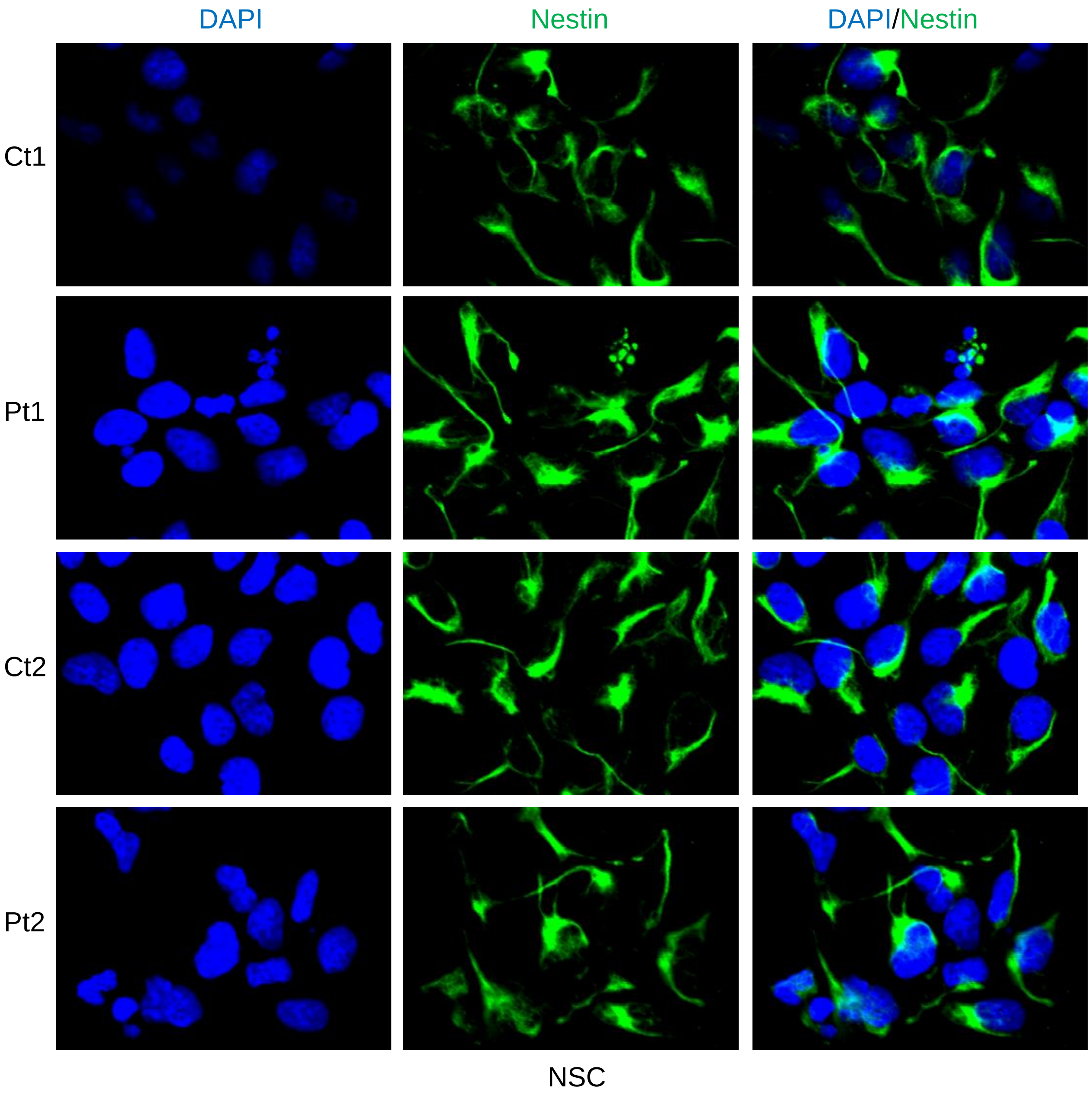

Fig. S1B

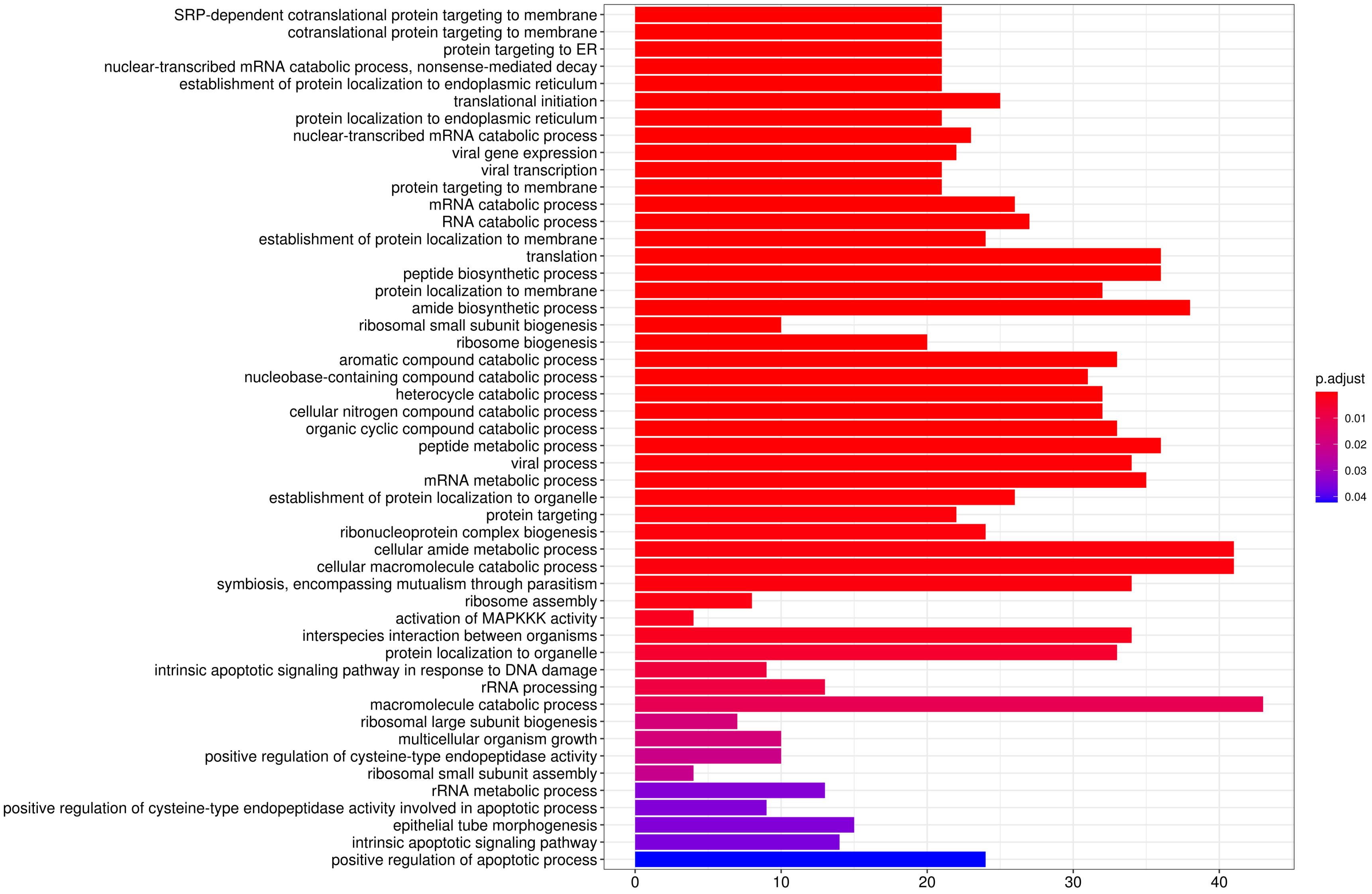
SRP-dependent cotranslational protein targeting to membrane
cotranslational protein targeting to membrane
protein targeting to ER
nuclear-transcribed mRNA catabolic process, nonsense-mediated decay
establishment of protein localization to endoplasmic reticulum
translational initiation
protein localization to endoplasmic reticulum
nuclear-transcribed mRNA catabolic process
viral gene expression
viral transcription
protein targeting to membrane
mRNA catabolic process
RNA catabolic process
establishment of protein localization to membrane
translation
peptide biosynthetic process
protein localization to membrane
amide biosynthetic process
ribosomal small subunit biogenesis
ribosome biogenesis
aromatic compound catabolic process
nucleobase-containing compound catabolic process
heterocycle catabolic process
cellular nitrogen compound catabolic process
organic cyclic compound catabolic process
peptide metabolic process
viral process
mRNA metabolic process
establishment of protein localization to organelle
protein targeting
ribonucleoprotein complex biogenesis
cellular amide metabolic process
cellular macromolecule catabolic process
symbiosis, encompassing mutualism through parasitism
ribosome assembly
activation of MAPKKK activity
interspecies interaction between organisms
protein localization to organelle
intrinsic apoptotic signaling pathway in response to DNA damage
rRNA processing
macromolecule catabolic process
ribosomal large subunit biogenesis
multicellular organism growth
positive regulation of cysteine-type endopeptidase activity
ribosomal small subunit assembly
rRNA metabolic process
positive regulation of cysteine-type endopeptidase activity involved in apoptotic process
epithelial tube morphogenesis
intrinsic apoptotic signaling pathway
positive regulation of apoptotic process
0
10
20
30
40
p.adjust
0.01
0.02
0.03
0.04

Fig. S2A

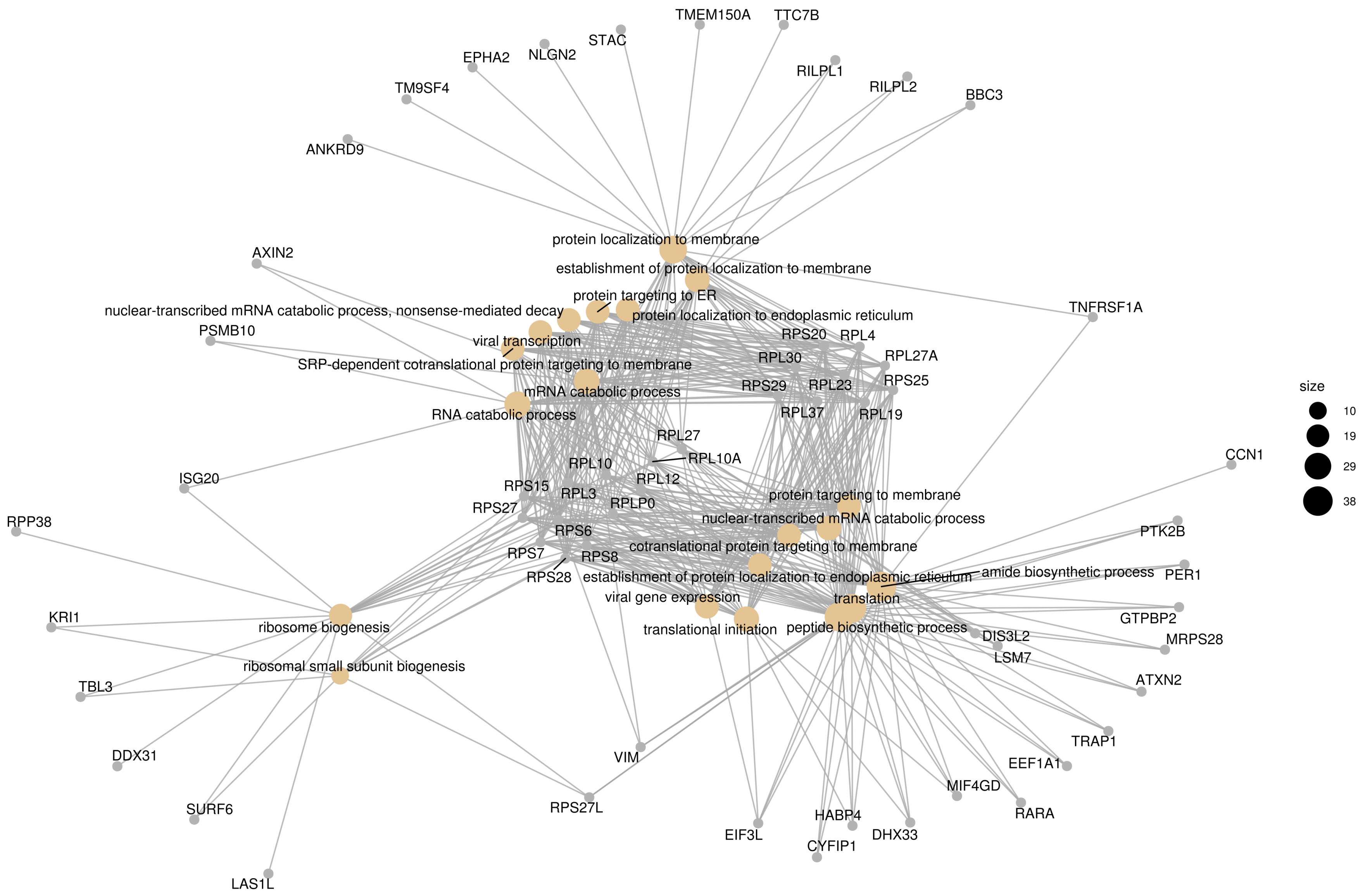
TMEM150A
TTC7B
STAC
NLGN2
EPHA2
RILPL1
RILPL2
BBC3
TM9SF4
ANKRD9
protein localization to membrane
establishment of protein localization to membrane
AXIN2
protein targeting to ER
nuclear-transcribed mRNA catabolic process, nonsense-mediated decay
protein localization to endoplasmic reticulum
TNFRSF1A
PSMB10
RPS20
RPL4
viral transcription
RPL27A
RPL30
SRP-dependent cotranslational protein targeting to membrane
RPS29
RPL23
RPS25
mRNA catabolic process
RNA catabolic process
RPL37
RPL19
RPL27
RPL10A
RPL10
ISG20
RPL12
RPS15
RPL3
RPLP0
protein targeting to membrane
RPS27
nuclear-transcribed mRNA catabolic process
RPP38
RPS6
RPS7
RPS8
cotranslational protein targeting to membrane
RPS28
establishment of protein localization to endoplasmic reticulum
amide biosynthetic process
viral gene expression
translation
KRI1
ribosome biogenesis
translational initiation
peptide biosynthetic process
ribosomal small subunit biogenesis
TBL3
DDX31
SURF6
LAS1L
RPS27L
VIM
EIF3L
CYFIP1
HABP4
DHX33
MIF4GD
RARA
EEF1A1
TRAP1
ATXN2
LSM7
DIS3L2
MRPS28
GTPBP2
PER1
PTK2B
CCN1
size
10
19
29
38

Fig. S2B

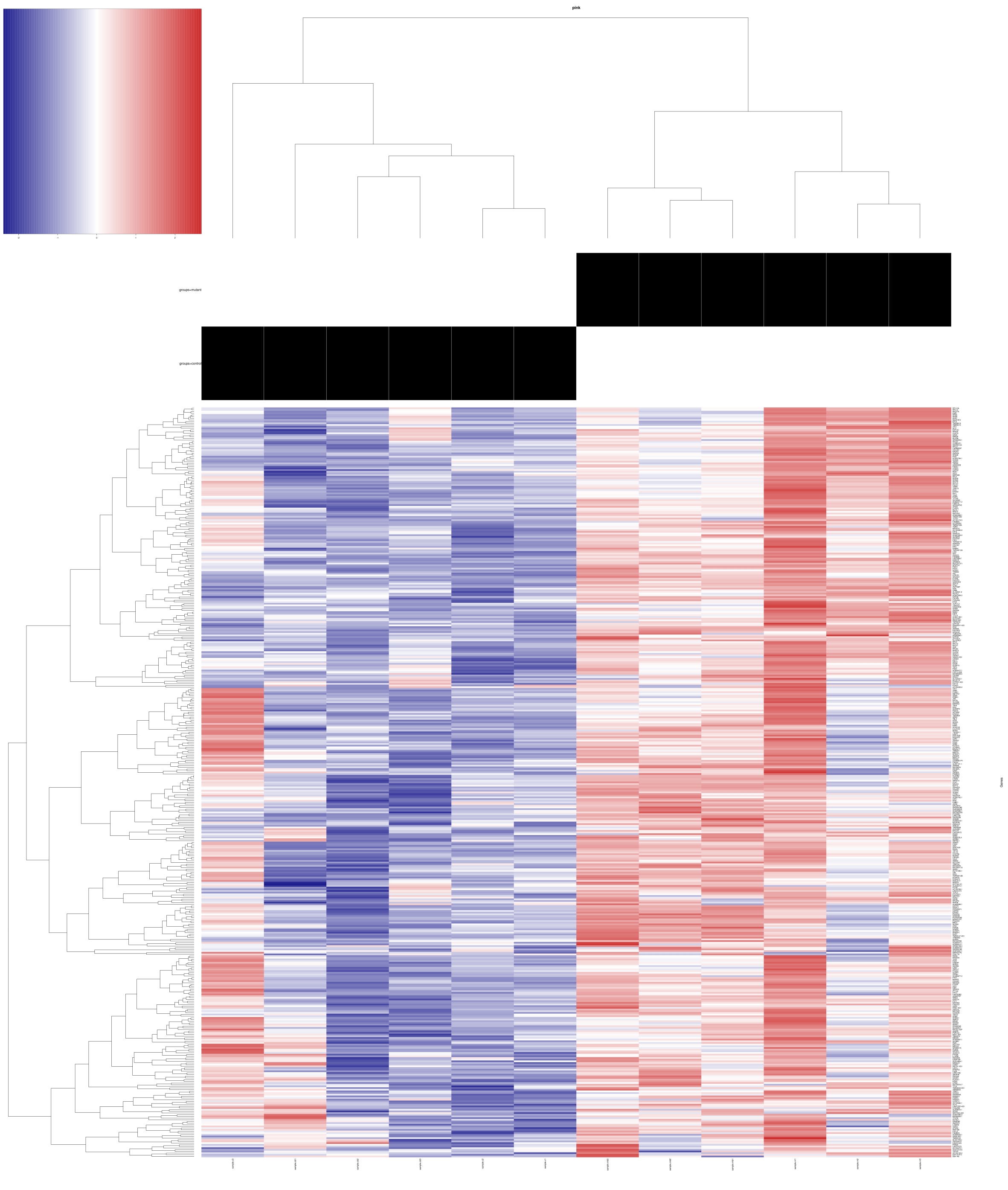

Fig. S2C

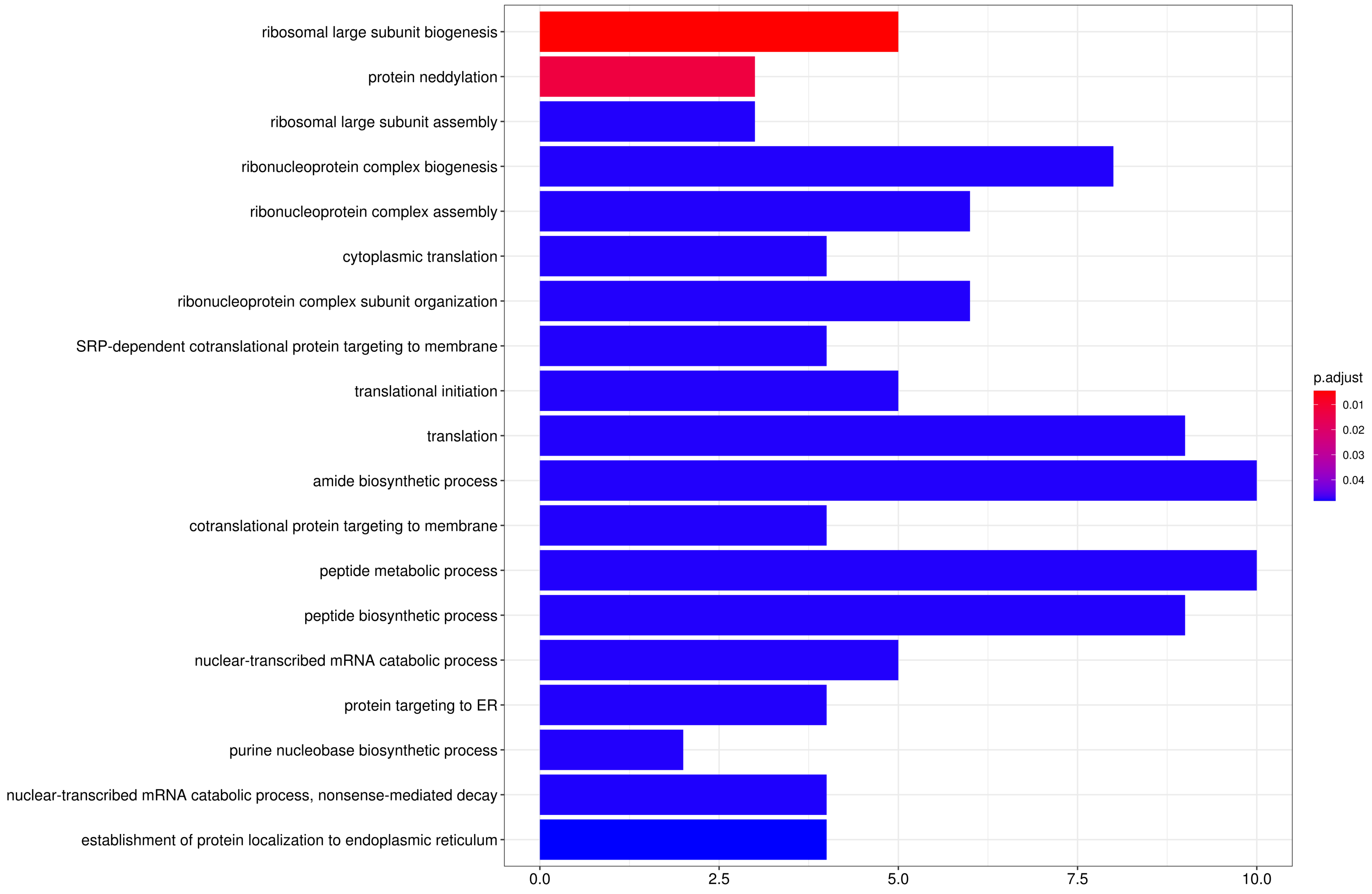
ribosomal large subunit biogenesis
protein neddylation
ribosomal large subunit assembly
ribonucleoprotein complex biogenesis
ribonucleoprotein complex assembly
cytoplasmic translation
ribonucleoprotein complex subunit organization
SRP-dependent cotranslational protein targeting to membrane
translational initiation
translation
amide biosynthetic process
cotranslational protein targeting to membrane
peptide metabolic process
peptide biosynthetic process
nuclear-transcribed mRNA catabolic process
protein targeting to ER
purine nucleobase biosynthetic process
nuclear-transcribed mRNA catabolic process, nonsense-mediated decay
establishment of protein localization to endoplasmic reticulum
0.0
2.5
5.0
7.5
10.0
p.adjust
0.01
0.02
0.03
0.04

Fig. S3A

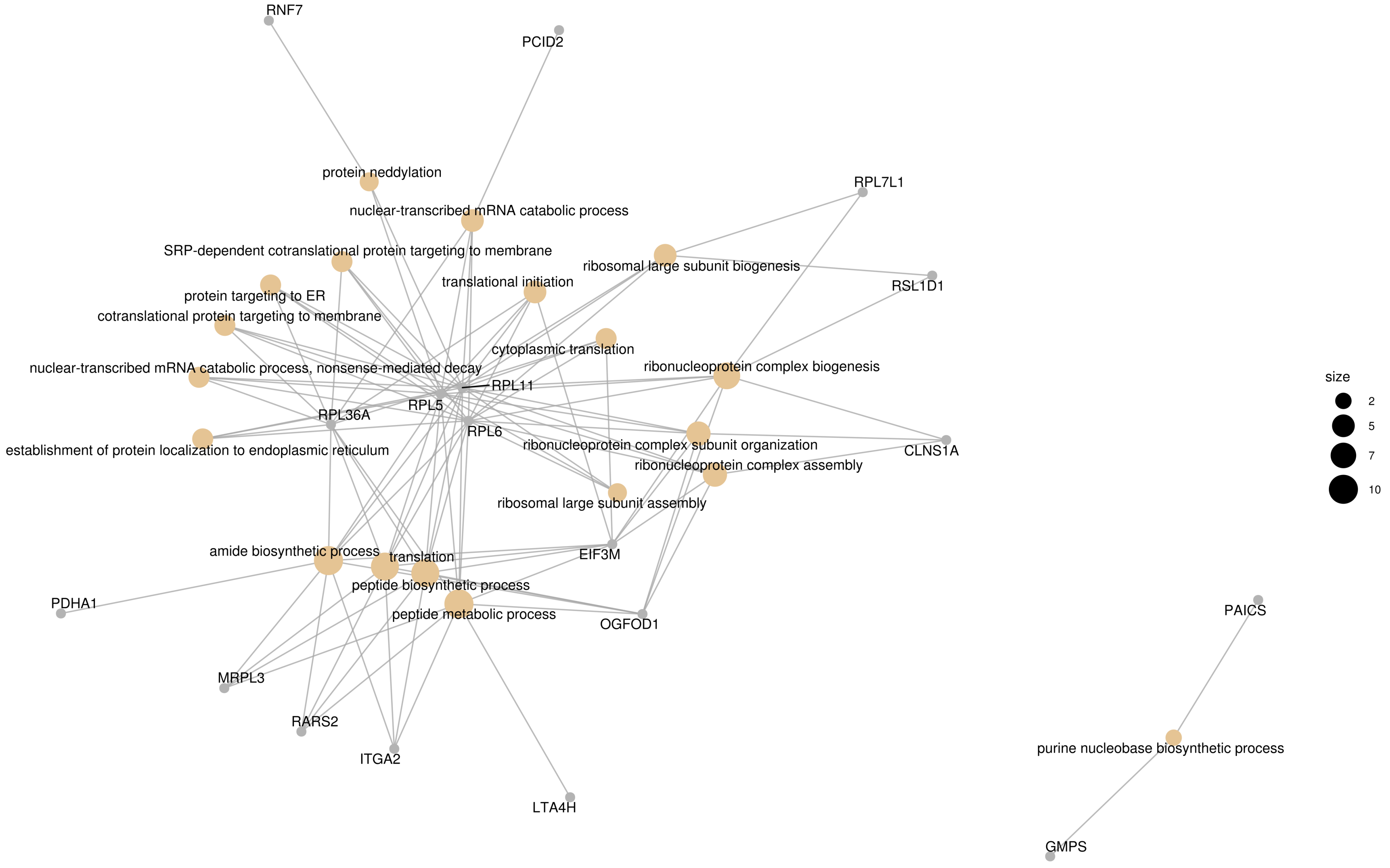
RNF7
PCID2
protein neddylation
nuclear-transcribed mRNA catabolic process
SRP-dependent cotranslational protein targeting to membrane
RPL7L1
ribosomal large subunit biogenesis
RSL1D1
translational initiation
protein targeting to ER
cotranslational protein targeting to membrane
cytoplasmic translation
nuclear-transcribed mRNA catabolic process, nonsense-mediated decay
ribonucleoprotein complex biogenesis
RPL11
RPL5
RPL36A
RPL6
establishment of protein localization to endoplasmic reticulum
ribonucleoprotein complex subunit organization
ribonucleoprotein complex assembly
CLNS1A
ribosomal large subunit assembly
amide biosynthetic process
translation
EIF3M
peptide biosynthetic process
peptide metabolic process
OGFOD1
PDHA1
MRPL3
RARS2
ITGA2
LTA4H
PAICS
purine nucleobase biosynthetic process
GMPS
size
2
5
7
10

Fig. S3B

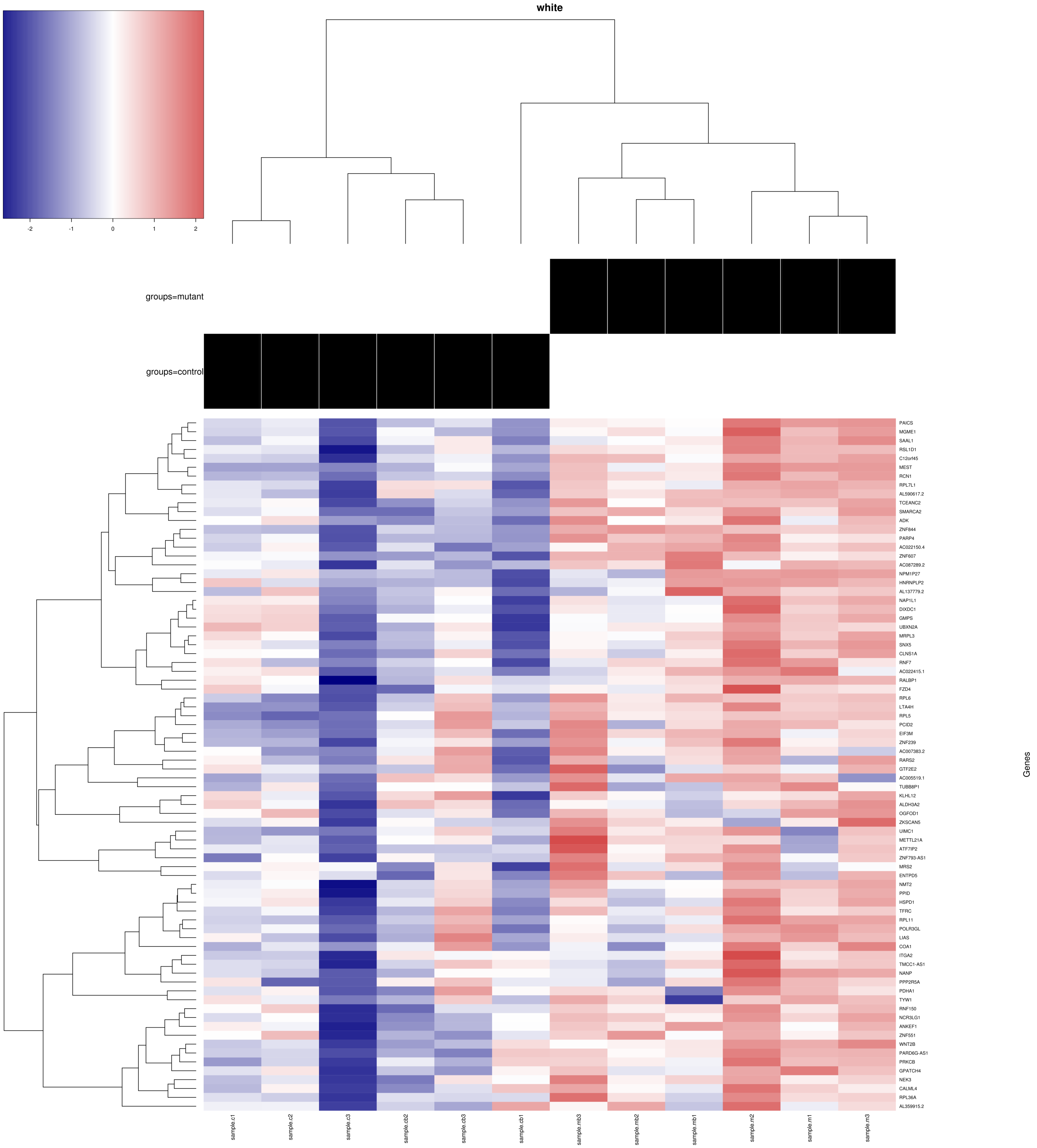

white
-2
-1
0
1
2
groups=mutant
groups=control
PAICS
MGME1
SAAL1
RSL1D1
C12orf45
MEST
RCN1
RPL7L1
AL590617.2
TCEANC2
SMARCA2
ADK
ZNF844
PARP4
AC022150.4
ZNF607
AC087289.2
NPM1P27
HNRNPLP2
AL137779.2
NAP1L1
DIXDC1
GMPS
UBXN2A
MRPL3
SNX5
CLNS1A
RNF7
AC022415.1
RALBP1
FZD4
RPL6
LTA4H
RPL5
PCID2
EIF3M
ZNF239
AC007383.2
RARS2
GTF2E2
AC005519.1
TUBB8P1
KLHL12
ALDH3A2
OGFOD1
ZKSCAN5
UIMC1
METTL21A
ATF7IP2
ZNF793-AS1
MRS2
ENTPD5
NMT2
PPID
HSPD1
TFRC
RPL11
POLR3GL
LIAS
COA1
ITGA2
TMCC1-AS1
NANP
PPP2R5A
PDHA1
TYW1
RNF150
NCR3LG1
ANKEF1
ZNF551
WNT2B
PARD6G-AS1
PRKCB
GPATCH4
NEK3
CALML4
RPL36A
AL359915.2
Genes
sample.c1
sample.c2
sample.c3
sample.cb2
sample.cb3
sample.cb1
sample.mb3
sample.mb2
sample.mb1
sample.m2
sample.m1
sample.m3

Fig. S3C

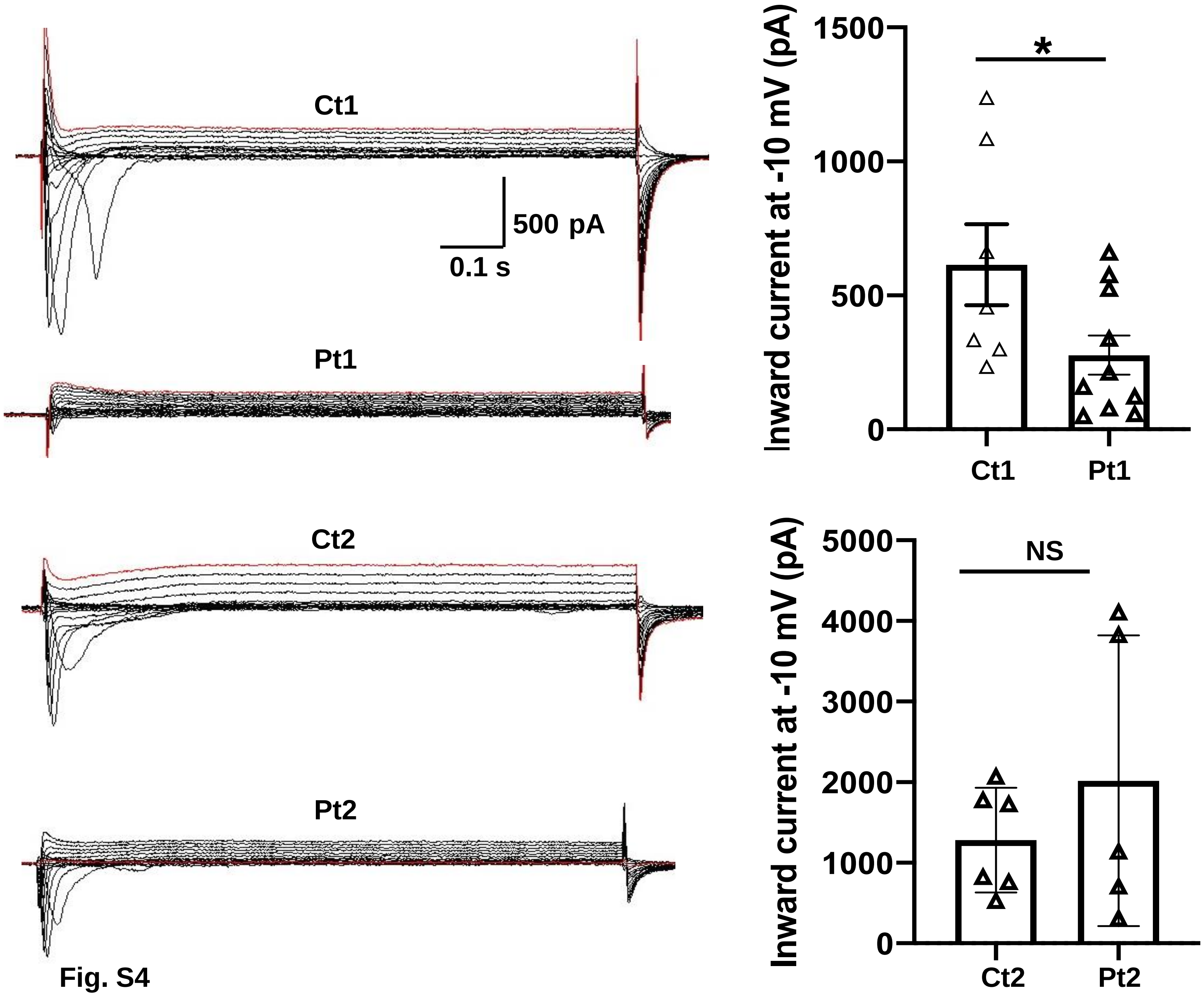
Ct1
500 pA
0.1 s
Pt1
Ct2
Pt2
Inward current at -10 mV (pA)
1500
1000
500
0
*
Ct1
Pt1
Inward current at -10 mV (pA)
5000
4000
3000
2000
1000
0
NS
Ct2
Pt2

Fig. S4
